# Mouse models of lung-specific SARS-CoV-2 infection with moderate pathological traits

**DOI:** 10.3389/fimmu.2022.1055811

**Published:** 2022-11-15

**Authors:** Sung-Hee Kim, Jiseon Kim, Ji Yun Jang, Hyuna Noh, Jisun Park, Haengdueng Jeong, Donghun Jeon, Chanyang Uhm, Heeju Oh, Kyungrae Cho, Yoon Jeon, Dain On, Suhyeon Yoon, Soo-Yeon Lim, Sol Pin Kim, Youn Woo Lee, Hui Jeong Jang, In Ho Park, Jooyeon Oh, Jung Seon Seo, Jeong Jin Kim, Sang-Hyuk Seok, Yu Jin Lee, Seung-Min Hong, Se-Hee An, Seo Yeon Kim, Young Been Kim, Ji-Yeon Hwang, Hyo-Jung Lee, Hong Bin Kim, Kang-Seuk Choi, Jun Won Park, Jun-Young Seo, Jun-Won Yun, Jeon-Soo Shin, Ho-Young Lee, Kyoungmi Kim, Daekee Lee, Ho Lee, Ki Taek Nam, Je Kyung Seong

**Affiliations:** ^1^ Severance Biomedical Science Institute, Graduate School of Medical Science, Brain Korea 21 Project, Yonsei University College of Medicine, Seoul, South Korea; ^2^ Division of Cancer Biology, Research Institute, National Cancer Center, Goyang, Gyeonggi, South Korea; ^3^ College of Pharmacy, Dongguk University, Seoul, South Korea; ^4^ Korea Mouse Phenotyping Center, Seoul National University, Seoul, South Korea; ^5^ Department of Life Science, Ewha Womans University, Seoul, South Korea; ^6^ Laboratory of Developmental Biology and Genomics, Research Institute for Veterinary Science, and BK21 PLUS Program for Creative Veterinary Science Research, College of Veterinary Medicine, Seoul National University, Seoul, South Korea; ^7^ Department of Nuclear Medicine, Seoul National University Bundang Hospital, Seongnam, South Korea; ^8^ Institute of Immunology and Immunological Diseases, Yonsei University College of Medicine, Seoul, South Korea; ^9^ Department of Microbiology, Yonsei University College of Medicine, Seoul, South Korea; ^10^ Division of Biomedical Convergence, College of Biomedical Science, Kangwon National University, Chuncheon, South Korea; ^11^ Laboratory of Avian Diseases, BK21 plus Program for Veterinary Science and Research Institute for Veterinary Science, College of Veterinary Medicine, Seoul National University, Seoul, South Korea; ^12^ Preclinical Research Center, Seoul National University Bundang Hospital, Seongnam, South Korea; ^13^ Department of Periodontology, Section of Dentistry, Seoul National University Bundang Hospital, Seongnam, South Korea; ^14^ Department of Internal Medicine, Seoul National University Bundang Hospital, Seoul National University College of Medicine, Seongnam, South Korea; ^15^ Laboratory of Veterinary Toxicology, College of Veterinary Medicine, Seoul National University, Seoul, South Korea; ^16^ Department of Nuclear Medicine, College of Medicine, Seoul National University, Seoul, South Korea; ^17^ Department of Physiology and Biomedical Science, Korea University College of Medicine, Seoul, South Korea; ^18^ Graduate School of Cancer Science and Policy, National Cancer Center, Goyang, Gyeonggi, South Korea; ^19^ BIO-MAX Institute, Seoul National University, Seoul, South Korea; ^20^ Interdisciplinary Program for Bioinformatics, Seoul National University, Seoul, South Korea

**Keywords:** SARS-CoV-2, hACE2 transgenic mice, K18-hACE2 mice model, SFTPB-hACE2 mice model, SCGB1A1-hACE2 mice model

## Abstract

Severe acute respiratory syndrome coronavirus 2 (SARS-CoV-2) causing coronavirus disease 2019 (COVID-19) has been a global health concern since 2019. The viral spike protein infects the host by binding to angiotensin-converting enzyme 2 (ACE2) expressed on the cell surface, which is then processed by type II transmembrane serine protease. However, ACE2 does not react to SARS-CoV-2 in inbred wild-type mice, which poses a challenge for preclinical research with animal models, necessitating a human ACE2 (hACE2)-expressing transgenic mouse model. Cytokeratin 18 (*K18*) promoter-derived hACE2 transgenic mice [B6.Cg-Tg(K18-ACE2)2Prlmn/J] are widely used for research on SARS-CoV-1, MERS-CoV, and SARS-CoV-2. However, SARS-CoV-2 infection is lethal at ≥10^5^ PFU and SARS-CoV-2 target cells are limited to type-1 alveolar pneumocytes in K18-hACE2 mice, making this model incompatible with infections in the human lung. Hence, we developed lung-specific SARS-CoV-2 infection mouse models with surfactant protein B (*SFTPB*) and secretoglobin family 1a member 1 (*Scgb1a1*) promoters. After inoculation of 10^5^ PFU of SARS-CoV-2 to the K18-hACE2, SFTPB-hACE2, and SCGB1A1-hACE2 models, the peak viral titer was detected at 2 days post-infection and then gradually decreased. In K18-hACE2 mice, the body temperature decreased by approximately 10°C, body weight decreased by over 20%, and the survival rate was reduced. However, SFTPB-hACE2 and SCGB1A1-hACE2 mice showed minimal clinical signs after infection. The virus targeted type I pneumocytes in K18-hACE2 mice; type II pneumocytes in SFTPB-hACE2 mice; and club, goblet, and ciliated cells in SCGB1A1-hACE2 mice. A time-dependent increase in severe lung lesions was detected in K18-hACE2 mice, whereas mild lesions developed in SFTPB-hACE2 and SCGB1A1-hACE2 mice. Spleen, small intestine, and brain lesions developed in K18-hACE2 mice but not in SFTPB-hACE2 and SCGB1A1-hACE2 mice. These newly developed SFTPB-hACE2 and SCGB1A1-hACE2 mice should prove useful to expand research on hACE2-mediated respiratory viruses.

## Introduction

Severe acute respiratory syndrome coronavirus-2 (SARS-CoV-2) causes the respiratory disease coronavirus disease 2019 (COVID-19), including pneumonia (1, 2). Since its emergence in China in December 2019, COVID-19 rapidly spread globally, which was declared a pandemic by the World Health Organization in March 2020. Over the two years, various strains of SARS-CoV-2 continue to spread worldwide (3, 4).

Human angiotensin-converting enzyme 2 (ACE2) plays a key role in SARS-CoV-2 infection, as the viral spike (S) protein uses ACE2 as a receptor to enter the host cell, which is then processed by type II transmembrane serine protease (TMPRSS2) (5, 6). ACE2-expressing type 2 alveolar pneumocytes, ciliated cells, and goblet cells are the main targets of SARS-CoV-2 in the human lungs (7). However, ACE2 of inbred wild-type mice does not react with SARS-CoV-2 S protein (8, 9), necessitating humanized transgenic mice expressing human (h)ACE2 to perform COVID-19–related research. Currently, the hACE2 transgenic mouse model with the cytokeratin 18 (*K18*) promoter [B6.Cg-Tg(K18-ACE2)2Prlmn/J] is widely used for this purpose, as these mice are susceptible to infection with SARS-CoV-1, Middle Eastern Respiratory Syndrome (MERS)-CoV, and SARS-CoV-2 (8, 10, 11).

However, an infectious dose of ≥10^4^ PFU of SARS-CoV-2 causes severe clinical symptoms in K18-hACE2, characterized by a gradual decrease in body weight, lung lesions, and high mortality at 5–7 days post-infection (dpi) (12–16). The virus is detected at higher levels in the lungs of K18-hACE2 mice than in other organs, such as the spleen, small intestine, and brain (13, 14, 17), which is likely attributed to *K18* promoter expression in the epithelial tissue. Moreover, SARS-CoV-2 target cells are limited to the type 1 alveolar pneumocytes in K18-hACE2 mice, which differs from the infectious targets of the human lung (18, 19).

Therefore, to provide a more suitable model for COVID-19–related research, the aim of this study was to develop new SARS-CoV-2 infection mouse models with lung-specific expression of hACE2 in various infectious target cells, besides for type 2 alveolar cells. For this purpose, we focused on the surfactant protein B (SFTPB) and secretoglobin family 1a member 1 (SCGB1A1) gene promoters. SFTPB is secreted by type 2 alveolar pneumocytes and non-ciliated bronchiolar cells in the lungs to maintain lung homeostasis (20–22). SCGB1A1 is also a pulmonary surfactant protein and marker of Clara cells, which is the predominant cell type in the airway epithelium (23–25). SCGB1A1 in bronchiole Clara cells plays an important role in lung inflammation and the immune response to respiratory syncytial virus infection (24, 25).

In this study, we evaluated the response of these hACE2-expressing transgenic mice driven by the *SFTPB* and *Scgb1a1* promoters to SARS-CoV-2 infection in comparison with that of K18-hACE2 mice. We performed an in-depth pathological analysis of various organs, including the lung, spleen, intestine, and brain, in the three hACE2 transgenic mouse models.

## Results

### SARS-CoV-2 infection is lethal in K18-hACE2 mice but not in SFTPB-hACE2 and SCGB1A1-hACE2 mice

**Figure 1:**
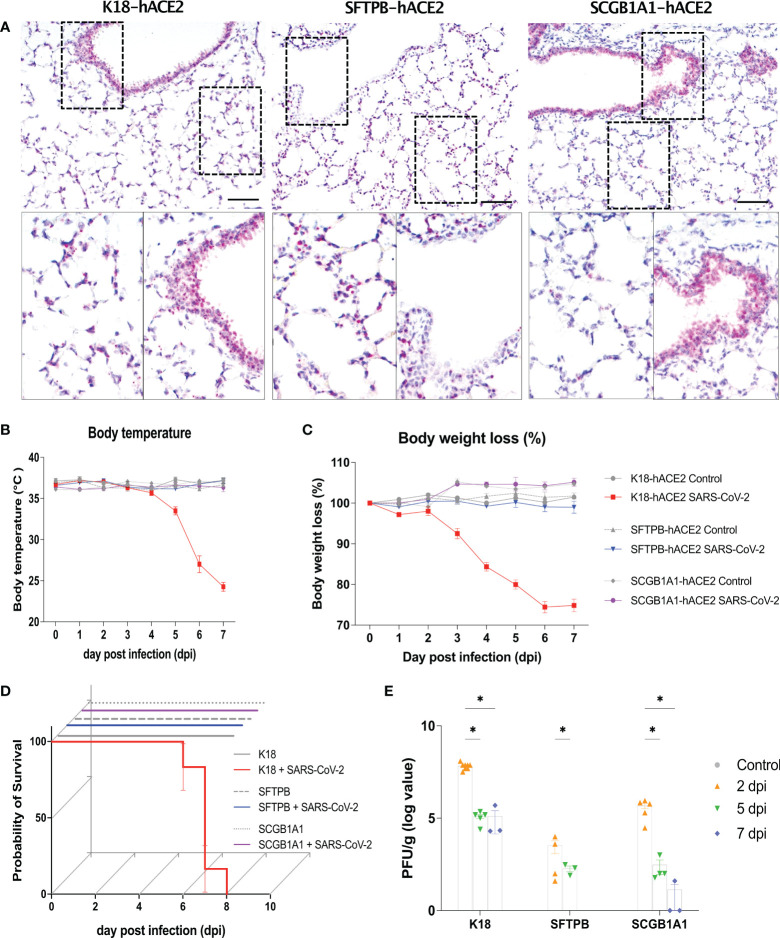
Human ACE2 expression in the lung and clinical parameters of SARS-CoV-2 infection in the K18-hACE2, SFTPB-hACE2, and SCGB1A1-hACE2 mouse models. **(A)** Human (h) ACE2 expression in the lung analysed by *in situ* hybridisation (ISH). Scale bar = 100 μm. **(B–D)** Preclinical parameters, body temperature, body weight loss, and survival rate, in animals infected with 1 × 10^5^ PFU of SARS-CoV-2 (K18-hACE2 and SFTPB-hACE2, n = 25; SCGB1A1-hACE2, n = 12) and non-infected control animals (K18-hACE2 and SFTPB-hACE2, n = 5; SCGB1A1-hACE2, n = 3) in each hACE2 transgenic mice model. **(E)** PFU (titre) measured in the lungs by the plaque assay. Data represent mean ± standard error. *P* values were obtained by two-tailed unpaired Student’s t-test (*P < 0.05).

We developed lung-specific SARS-CoV-2 infectious mouse models that expressed the human *ACE2* gene using *SFTPB* and *Scgb1a1* promoters (**Supplementary Figures 1A, B**). *In situ* hybridisation confirmed hACE2 expression in the three transgenic mouse models (SFTPB-hACE2, SCGB1A1-hACE2, and K18-hACE2 mice). hACE2 expression was detected in the type I alveolar cells and bronchos region of K18-hACE2 mice, in contrast to the lack of expression in wild-type C57BL/6 mice, whereas hACE2 was detected in the alveolar region of SFTPB-hACE2 mice and in the bronchos region of SCGB1A1-hACE2 mice, in contrast to the wild-type FVB/NJ mice (**Figure 1A**, **Supplementary Figures 1C, D**).

After infection of K18-hACE2 mice with SARS-CoV-2, the body temperature gradually decreased by approximately 10°C and over 20% body weight loss was observed compared with those of mock-infected K18-hACE2 mice until 7 dpi. However, no such changes were observed in SARS-CoV-2-infected SFTPB-hACE2 and SCGB1A1-hACE2 mice (**Figures 1B, C**). Survival rate was correlated with body temperature and weight loss; SARS-CoV-2 infection was lethal to K18-hACE2 mice but not to SFTPB-hACE2 and SCGB1A1-hACE2 mice (**Figure 1D**).

The viral titre was measured as of 2 dpi, with the highest PFU detected at 2 dpi, which then gradually significantly decreased over time in all hACE2 transgenic mice (**Figure 1E**). Compared with those of the control mice, the liver weight decreased (P < 0.005) and the brain weight increased (P < 0.05) in SARS-CoV-2-infected K18-hACE2 mice at 7 dpi (**Supplementary Figure 2**). The liver weight also increased at 1 and 7 dpi in SCGB1A1-hACE2 mice but decreased at 5 dpi. However, there were no changes in the organ weights of SARS-CoV-2-infected SFTPB-hACE2 mice.

Collectively, these data demonstrated that SARS-CoV-2 infection causes high mortality in K18-hACE2 mice, accompanied by body temperature and weight loss, but shows only rare and minor clinical signs in SFTPB-hACE2 and SCGB1A1-hACE2 mice.

### SARS-CoV-2 induces mild lung lesions in SFTPB-hACE2 and SCGB1A1-hACE2 mice and severe lung lesions in K18-hACE2 mice

**Figure 2:**
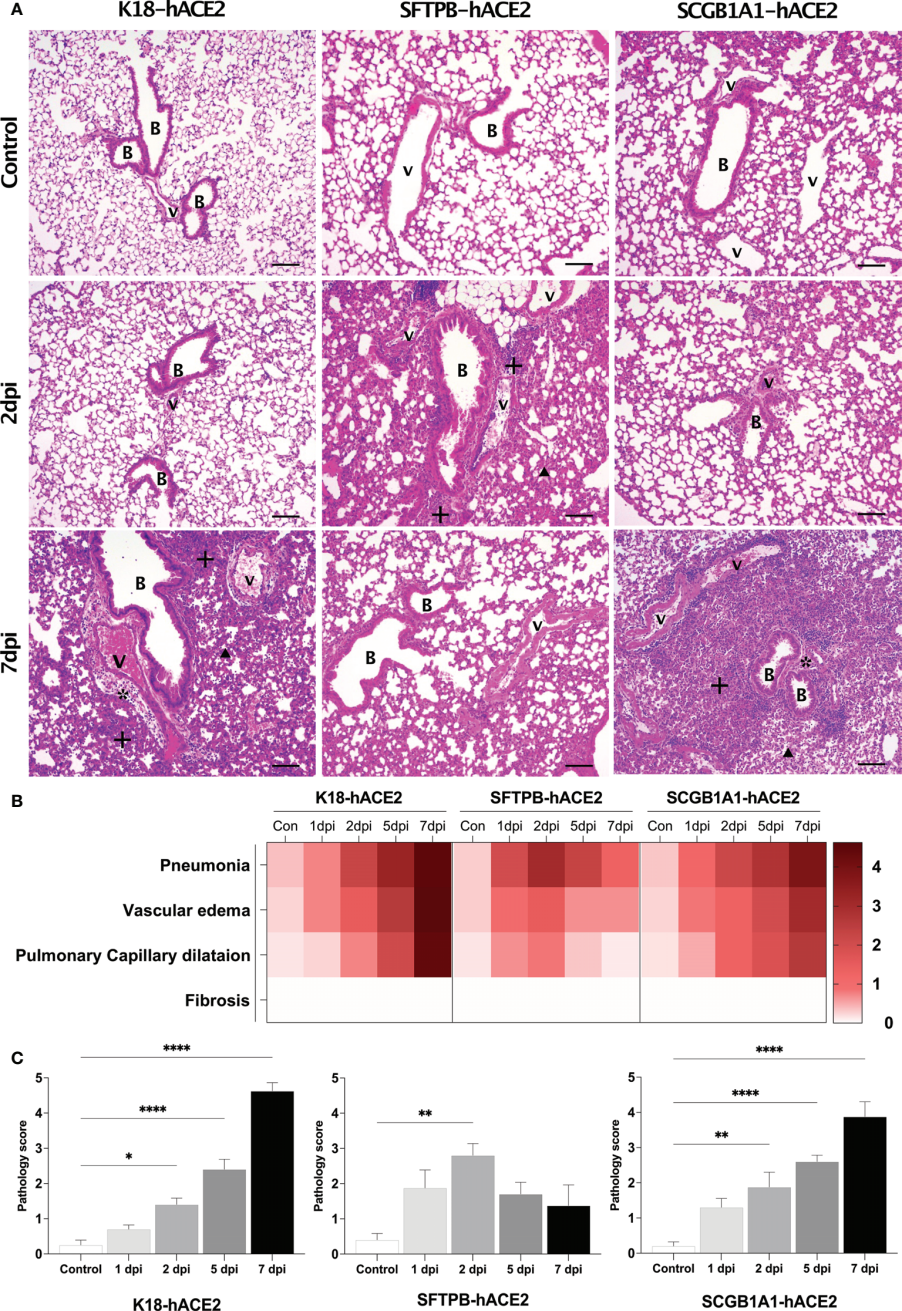
Histopathological analysis of SARS-CoV-2-infected lungs in K18-hACE2, SFTPB-hACE2, and SCGB1A1-hACE2 mice. **(A)** H&E staining of the lungs in each mouse model following mock infection (control, top panels) or intranasal infection with 1 × 10^5^ PFU SARS-CoV-2 at 2 dpi (middle panels) and 7 dpi (bottom panels). Infected lungs showed pneumonia (+), vascular oedema (*), and pulmonary capillary dilatation (arrowhead). The scale bars are 100 μm; B, bronchiole; V, vessel. **(B)** Heatmap showing the histopathological parameters and the average scores for the lung in each mouse model at 1, 2, 5, and 7 dpi, and in the non-infected (control) mice. The severity score ranges from 0 to 5 (0 = none; 1 = weak; 2 = mild; 3 = moderate; 4 = severe; 5; = markedly severe). **(C)** Lung pathology scoring of SARS-CoV-2-infected mice at 1, 2, 5, and 7 dpi. (*P < 0.05; ** *P* < 0.01; **** *P* < 0.0001).

Given the different expression patterns of hACE2 in the three mouse models (**Figure 1A**), we hypothesised that each model has distinct pathogenic features and different SARS-CoV-2 target cells. Thus, in-depth pathological analysis of the SARS-CoV-2-infected mice was performed in samples obtained at 1, 2, 5, and 7 dpi. H&E staining showed progression of lung disease following SARS-CoV-2 infection in K18-hACE2 mice over time (**Figure 2**; **Supplementary Figure 3**). Immune cell infiltration from the blood vessels was detected in the lungs at 1 dpi, followed by gradual development of oedema around the blood vessels (**Figure 2A**, asterisk; **Supplementary Figure 3**). Subsequently, capillary dilatation was detected at the inflammation lesion (**Figure 2A**, arrowhead). At 7 dpi, there was an extensively damaged area with greater thickness of the alveolar septa due to infiltration of immune cells between alveolar cells. The pathological diagnosis was confirmed in each lobe, and in the upper and lower regions of the lung in all K18-hACE2 mice.

By contrast, in SFTPB-hACE2 mice, severe SARS-CoV-2-related lesions were observed at the early stage of infection, at 1–2 dpi (**Figure 2A**; **Supplementary Figure 4**). At 1 dpi, there was severe inflammation derived from immune cell infiltration near the blood vessels and bronchioles. Inflammation close to the blood vessels along with capillary dilatation in the alveolar region were prevalent at 2 dpi. However, there was a progressive tendency for the lesions to be alleviated at 5 and 7 dpi (**Figures 2B, C**). Neither oedema nor fibrosis was observed at any period of SARS-CoV-2 infection in SFTPB-hACE2 mice.

SCGB1A1-hACE2 mice showed a similar progressive pattern of lung lesion development to that of K18-hACE2 mice, with a time-dependent severity increase following SARS-CoV-2 infection (**Figure 2A**, arrow; **Supplementary Figure 5**). Immune cell infiltration was detected in the peripheral bronchiole and blood vessels, and oedema developed at 1 dpi in SCGB1A1-ACE2 mice. Subsequently, inflammation and capillary dilatation steadily increased until 7 dpi. Although the SARS-CoV-2-related lesions of SCGB1A1-hACE2 mice progressed over time, the severity was relatively moderate compared with that of the SARS-CoV-2-infected K18-hACE2 mice (**Figures 2B, C**).

**Figure 3:**
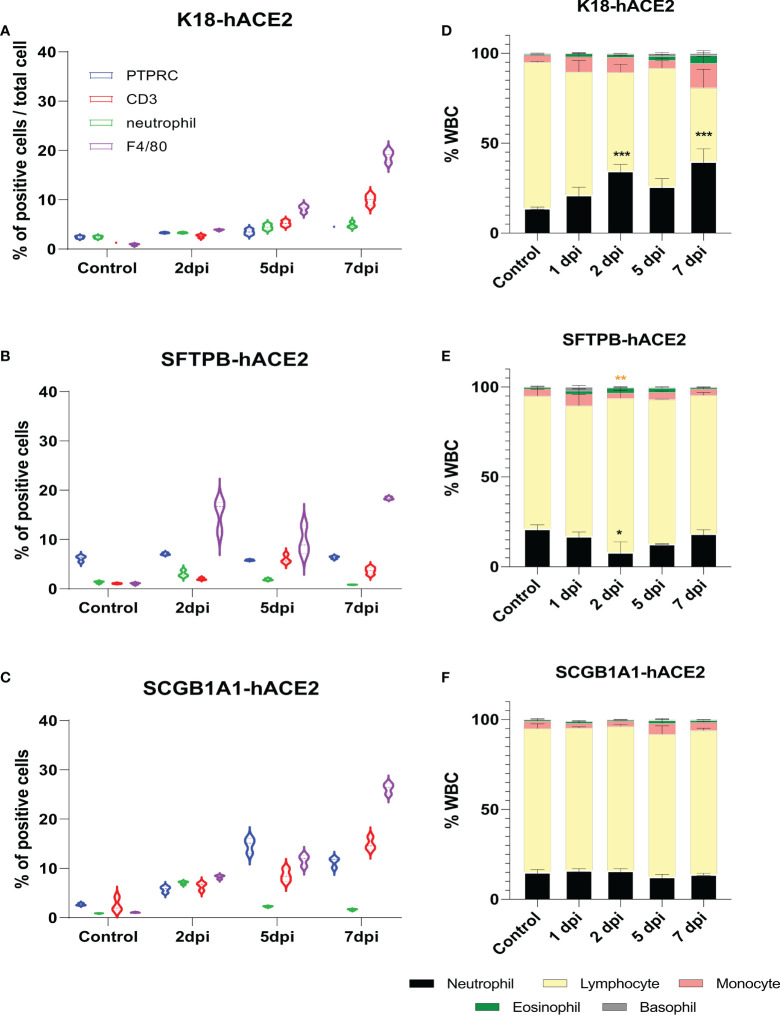
Change of immune cell distribution after SARS-CoV-2 infection in the lungs and peripheral blood. **(A–C)** Summary of the percentage of lung-infiltrated immune cells in K18-hACE2 **(A)**, SFTPB-hACE2 **(B)**, and SCGB1A1-hACE2 **(C)** mice. Immunohistochemistry was performed to identify the distribution of immune cells in the lungs with anti-PTPRC, CD3, neutrophil, and F4/80 antibodies at 0, 2, 5, and 7 dpi. DAB-positive cells were counted using QuPath and analysed from three randomly selected images. **(D–F)** White blood cell counts in K18-hACE2 **(D)**, SFTPB-hACE2 **(E)**, and SCGB1A1-hACE2 **(F)** mice from the peripheral blood at each time point post-infection.

Since immune cell dynamics, including neutrophil accumulation, is a characteristic feature in patients with COVID-19 pneumonia (26–29), we performed IHC for macrophage and neutrophil markers as well as two inflammatory lymphoid cell markers, PTPRC and CD3, to confirm the lung-infiltrated immune cells in each mouse model. There was accumulation of innate and adoptive immune response cells, especially F4/80^+^ macrophages and CD3^+^ T cells, over time in SARS-CoV-2-infected K18-hACE2 mice (**Figure 3A**; **Supplementary Figure 4**). SARS-CoV-2-infected SFTPB-hACE2 mice showed drastic infiltration of F4/80^+^ macrophages and a mild increase of neutrophils at 2 dpi, which subsequently decreased to 38.3% and 26.9% neutrophils and 36.9% and 62.2% F4/80^+^ macrophages at 5 and 7 dpi, respectively (**Figure 3B**; **Supplementary Figure 4**). In addition, CD3^+^ T cells temporally increased at 5 dpi and there was no change in PTPRC^+^ B cells throughout the infection period. In SCGB1A1-hACE2 mice, SARS-CoV-2 infection increased the infiltration of macrophages and CD3^+^ T cells from 2 to 7 dpi. Neutrophils transiently increased at 2 dpi, and PTPRC^+^ B cells increased at 2 and 5 dpi and then decreased at 7 dpi (**Figure 3C**; **Supplementary Figure 4**).

The neutrophil-to-lymphocyte (NTL) ratio is crucial for prognosis and mortality in patients with COVID-19 (29–35); thus, we hypothesised that the composition of peripheral blood leucocytes would also vary in the SARS-CoV-2 infection mouse models. CBC analysis showed that the NTL ratio significantly increased to 7.34%, 20.64%, 11.94%, and 25.01% at 1, 2, 5, and 7 dpi, respectively, in K18-hACE2 mice (**Figure 3D**). However, the NTL ratio did not substantially change in SARS-CoV-2-infected SFTPB-hACE2 and SCGB1A1-hACE2 mice (**Figures 3E, F**).

### SARS-CoV-2 targets different lung cell types in the three models

**Figure 4:**
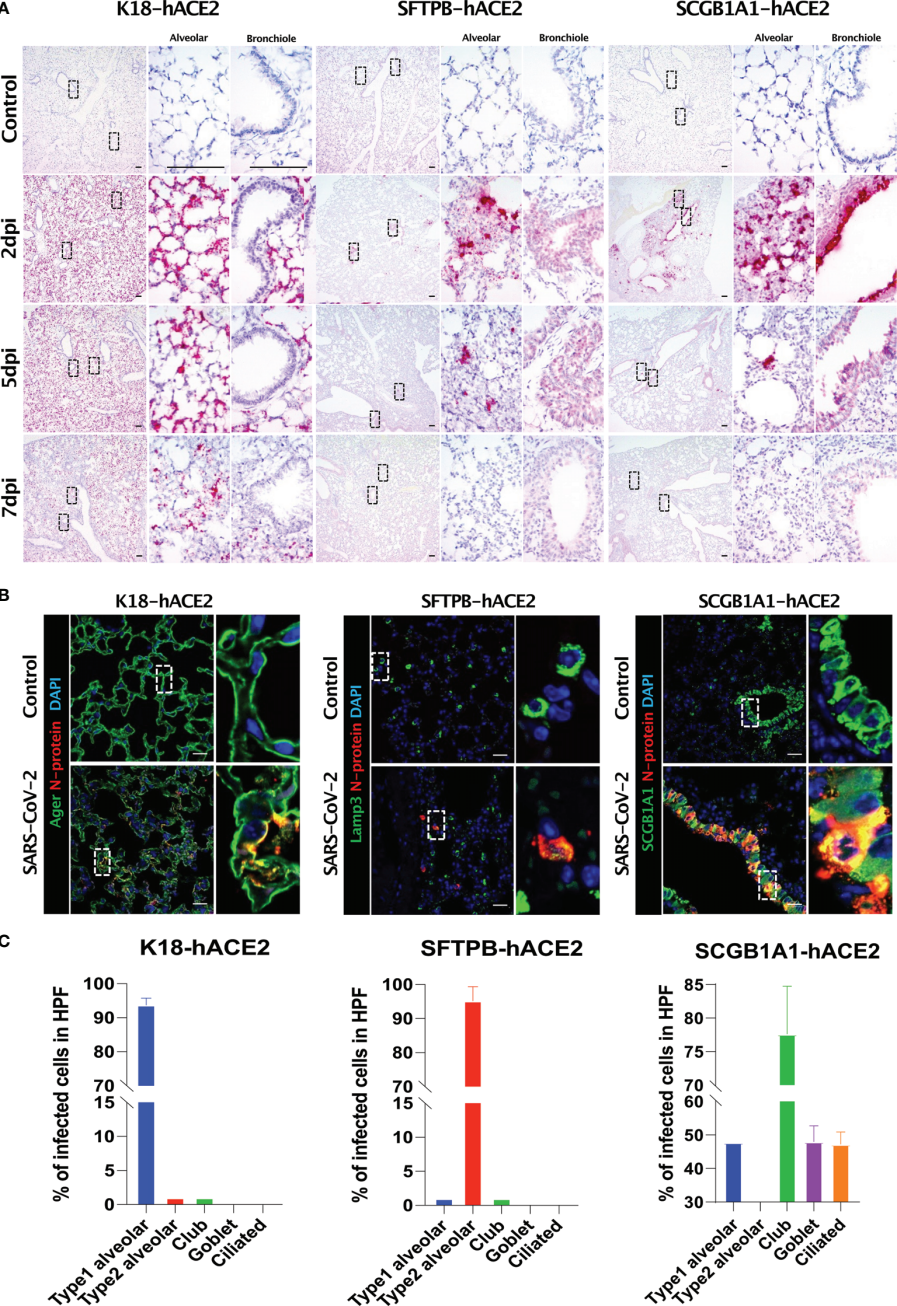
SARS-CoV-2 distribution and viral target cells in the lungs from each mouse model. **(A)** SARS-CoV-2 distribution in the lungs analysed using *in situ* hybridisation at 2, 5, and 7 dpi with 1 × 10^5^ PFU SARS-CoV-2. **(B)** Representative confocal immunofluorescence micrographs of the lungs of K18-hACE2 (left), SFTPB-hACE2 (middle), and SCGB1A1-hACE2 (right) mice from the PBS-treated control group (top) and SARS-CoV-2-infected group (bottom) at 2 dpi. SARS-CoV-2 N-protein-positive cells are stained red, and type 1 alveolar (Ager), type 2 alveolar (Lamp3), or club (SCGB1A1) cells are stained green. Nuclei of cells are stained blue with DAPI. The scale bars are 100 μm. **(C)** Quantification of the percentage of infected cells from the lungs of K18-hACE2 (left), SFTPB-hACE2 (middle), and SCGB1A1-hACE2 (right) mice analysed from the confocal immunofluorescence micrographs shown in **(B)**. Lung sections from SARS-CoV-2-infected mice at 2 dpi stained with anti-SARS-CoV-2 N-protein antibody and one of anti-Ager, anti-Lamp3, anti-SCGB1A1, anti-Muc5ac, or anti-tubulin antibodies.

*In situ* hybridisation of the *S* gene was performed to verify the primary infection site and viral distribution in the three hACE2 transgenic mouse models following SARS-CoV-2 infection (**Figure 4A**). The *S* gene was diffused in the alveolar region, and not in the bronchiole, in K18-hACE2 and SFTPB-hACE2 mice, but exhibited a local distribution in SFTPB-hACE2 mice (**Figure 4A**, 2 dpi). In SCGB1A1-hACE2 mice, the *S* gene was detected in both the alveolar and bronchiole regions, demonstrating different regions of primary SARS-CoV-2 infection in the three models. However, the *S* gene was detected at the highest level at 2 dpi and then gradually decreased until 7 dpi in all models.

We further investigated the specific cell types that SARS-CoV-2 targets using various pulmonary epithelial cell lineage markers (**Figures 4B, C**). In K18-hACE2 mice, 93.71% of AGER-expressing type I pneumocytes co-stained with SARS-CoV-2 N protein at 2 dpi, whereas 95.21% of LAMP3-expressing type II pneumocytes co-stained with N protein at 2 dpi in STTPB-hACE2 mice (**Supplementary Figure 5**). In SCGB1A1-hACE2 mice, N protein co-stained with SCGB1A1 (77.6%), MUC5AC (47.98%), and acetylated tubulin (47.23%), representing a bronchiole club cell marker, goblet cell marker, and ciliated cell marker, respectively (**Supplementary Figure 5**). However, chromogranin A-positive pulmonary neuroendocrine cells did not express N protein in any mouse model (**Supplementary Figure 5**). In addition, TEM showed that SARS-CoV-2 targeted type I pneumocytes in K18-hACE2 mice and ciliated cells in SCGB1A1-hACE2 mice (**Supplementary Figure 6**).

### SARS-CoV-2 infection causes damage to the spleen, small intestine, and brain in K18-hACE2 mice

Given that SARS-CoV-2 induced changes to the immune cell profiles of the mouse models with different degrees of pathological severity in the lung and a varied immune cell composition in the peripheral blood, we expected that SARS-CoV-2 infection could influence organs other than the lungs, as observed in human cases and mouse models (12, 36–39).

**Figure 5:**
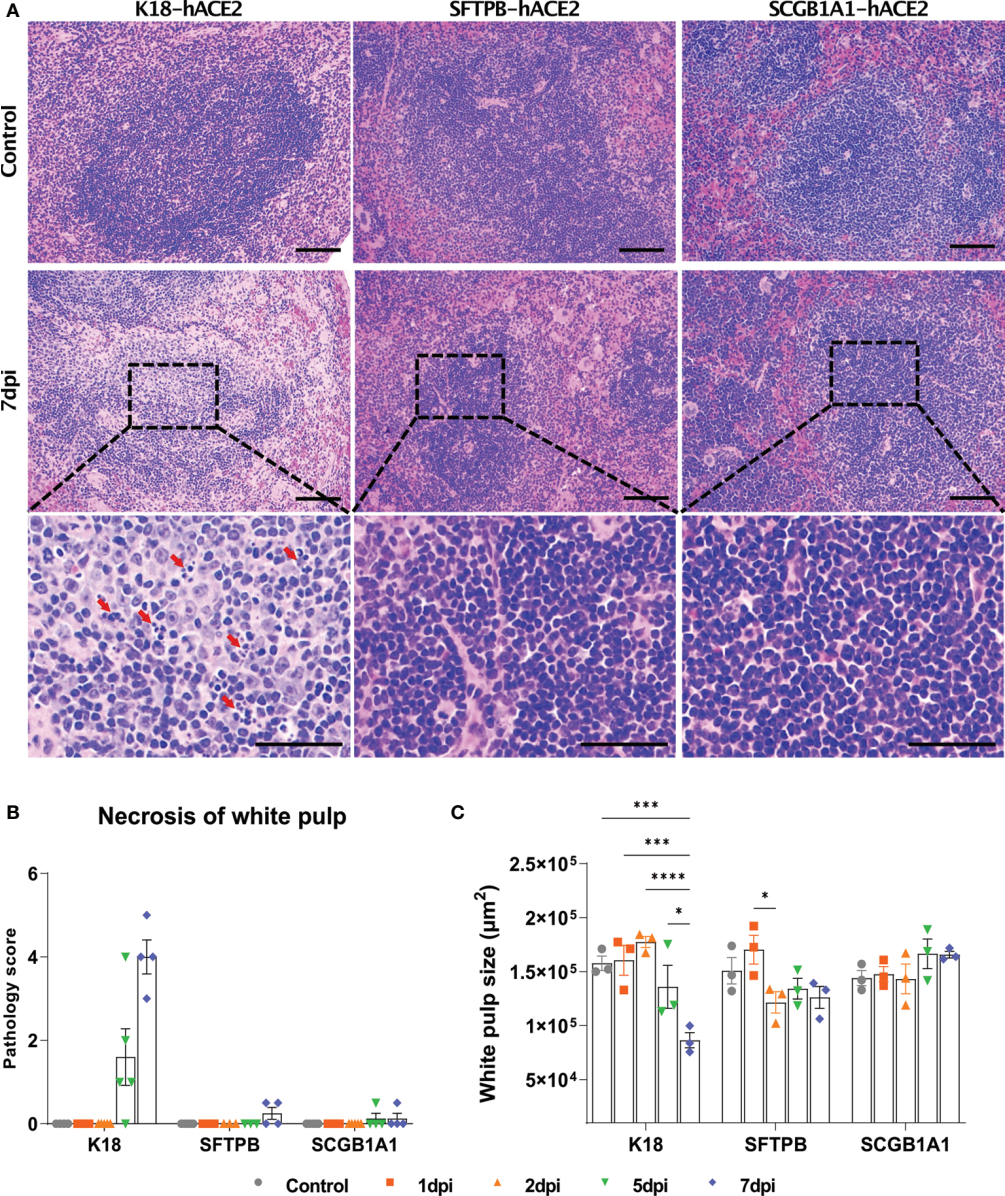
Pathological analysis and scoring of the spleen in SARS-CoV-2-infected K18-hACE2, SFTPB-hACE2, and SCGB1A1-hACE2 mice. **(A)** H&E staining of the spleen following injection with PBS (control, top panels) or 1 × 10^5^ PFU SARS-CoV-2. SARS-CoV-2 at 7 dpi (middle panels) and enlarged image (bottom panels). The scale bars are 100 μm (top and middle panels) and 50 μm (bottom panels). **(B)** Spleen pathology scoring of SARS-CoV-2-infected animals and mock-infected controls at 1, 2, 5, and 7 dpi. The severity score ranges from 0 to 5 (0 = none; 1 = weak; 2 = mild; 3 = moderate; 4 = severe; 5 = markedly severe). **(C)** White pulp size of SARS-CoV-2 infected with each animal in the non-infected control at 1, 2, 5, and 7 dpi. (* *P* < 0.05; *** *P* < 0.001; **** *P* < 0.0001).

Since the spleen is the largest secondary lymphoid organ, we performed equivalent pathological analysis and immune cell profiling of the spleen as in the lungs. In K18-hACE2 mice, the lesion caused by SARS-CoV-2 developed white pulp necrosis, with the pulp size decreasing over time (**Figures 5A and B**, **Supplementary Figure 7**). We also observed dramatic structural changes, such as increased T cell- and macrophage-positive areas, and a relative decreased B cell-positive area at 1 dpi (**Supplementary Figure 8**). After 5 dpi, the T cell-positive area recovered to the control level. By contrast, white pulp necrosis and structural changes of the spleen were rarely detected in SARS-CoV-2-infected SFTPB-hACE2 and SCGB1A1-hACE2 mice (**Figure 5**).

**Figure 6:**
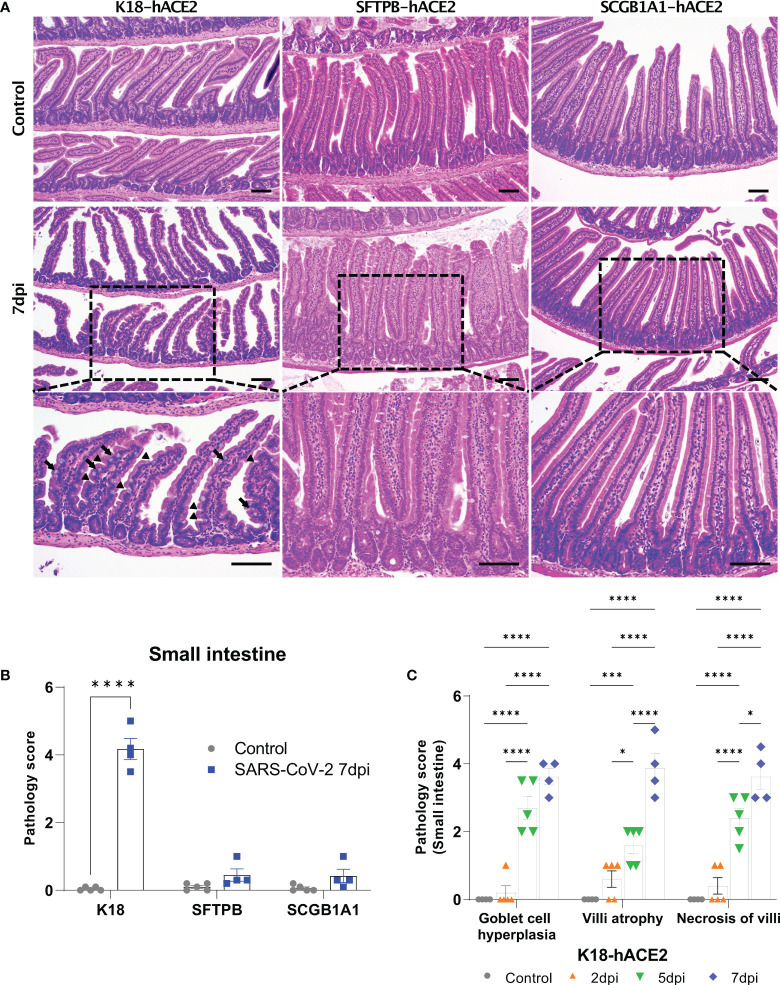
Pathological analysis of the small intestine in SARS-CoV-2-infected mice. **(A)** H&E staining of the small intestine in each mouse model following injection with PBS (control, top panels) or 1 × 10^5^ PFU SARS-CoV-2 infection (middle and bottom panels) at 7 dpi. The arrow shows goblet cell hyperplasia and the arrowhead shows villi atrophy. Scale bars =100 μm. **(B)** Total small intestinal pathology scoring of control and SARS-CoV-2-infected mice at 7 dpi. The severity score ranges from 0 to 5 (0 = none; 1 = weak; 2 = mild; 3 = moderate; 4 = severe; 5; = markedly severe). **(C)** Pathological scoring by lesion criteria of SARS-CoV-2-infected K18-hACE2 mice. (* *P* < 0.05; *** *P* < 0.001; **** P < 0.001).

**Figure 7:**
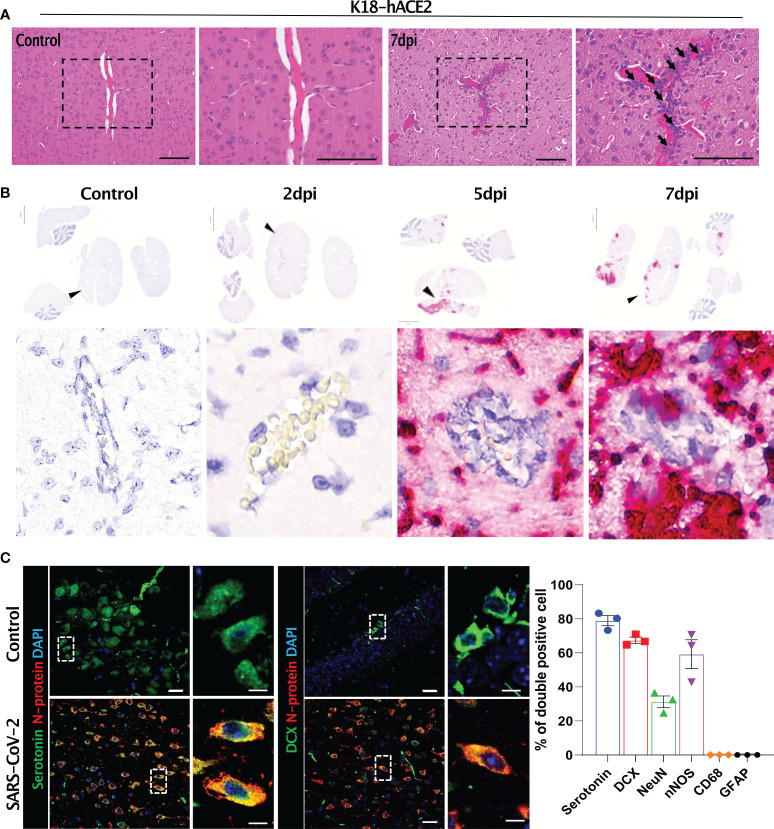
Histopathological features of the SARS-CoV-2-infected brain and its target cells in K18-hACE2 mice. **(A)** H&E-stained brain sections of control and infected mice. Black arrows with tails show perivascular cuffing. **(B)** *In situ* hybridisation on the sections in **(A)** for detecting the SARS-CoV-2 S gene. Black arrowheads indicate perivascular cuffing in *S* gene-positive infected lesions. **(C)** Representative images of immunofluorescence staining. Sections at 7 dpi were stained with anti-SARS-CoV-2 N protein and either anti-serotonin or anti-DCX antibody and other brain cell markers. Right, summary of the percentage of double-positive cells (SARS-CoV-2 N protein and each brain cell marker). Left panels: green (serotonin), red (N protein), blue (DAPI); right panels: green (DCX), red (N protein), blue (DAPI). Scale bars = 20 μm, 5 μm.

The SARS-CoV-2-related lesions of the small intestine, goblet cell hyperplasia, villi atrophy, and villi necrosis were detected only in K18-hACE2 mice, and the pathological score increased over time (**Figure 6**). Additionally multifocal perivascular cuffing, brain lesions, and SARS-CoV-2 *S* gene distribution were observed in K18-hACE2 mice after 5 dpi (**Figure 7**). In the brains of K18-hACE2 mice, 31.2% of NeuN^+^ cells, 79% of serotonin^+^ cells, 59.3% of nNOS^+^ cells, and 67.4% of DCX^+^ cells co-stained with SARS-CoV-2 N protein (**Figure 7C**), whereas CD68^+^ microglial cells and GFAP^+^ astrocytes did not express N protein (**Supplementary Figure 9**).

## Discussion

The K18-hACE2 transgenic mouse model expressing human ACE2 protein has been widely used in the study of SARS-CoV-1, MERS-CoV, and SARS-CoV-2; however, SARS-CoV or SARS-CoV-2 infection is lethal to K18-hACE2 mice, and the virus rapidly infects various organs, including the brain and spleen (8, 9, 12, 13, 17). Besides the poor survival rate, which poses a challenge for research, the SARS-CoV-2 infectious target lung cell is limited to type 1 alveolar pneumocytes in K18-hACE2 mice, which differs from the infection target in the human lungs. Thus, we generated a transgenic mouse with lung-specific expression of hACE2 using the promoters of SFTPB, a type 2 alveolar pneumocyte marker, and SCGB1A1, a Clara cell marker, and performed comparative pathological analysis after experimental SARS-CoV-2 infection in the three models.

SARS-CoV-2 targeted distinct lung cell types in the three models: type I alveolar cells in K18-hACE2 mice, type II alveolar cells in SFTPB-hACE2 mice, and bronchial cells in SCGB1A1-hACE2 mice. Moreover, the models showed distinct clinical signs and pathological traits of diverse organs following SARS-CoV-2 infection. In K18-hACE2 mice, intranasally administered SARS-CoV-2 rapidly spread to the whole body; thus, lesions were observed in various organs, especially in the lung, spleen, intestine, and brain. From 1 dpi, inflammation was observed around the blood vessels, and oedema, alveolar thickening, and capillary dilatation lesions increased over time. However, fibrosis was not detected in any SARS-CoV-2-infected K18-hACE2 mouse, which may be due to the lethality of the infection before fibrosis could be induced, as pulmonary fibrosis is the end stage of lung injury caused by various agents, viral infection, and other insults (40, 41). The body weight and body temperature of K18-hACE2 mice continuously decreased by approximately 10°C and 20%, respectively, until 7 dpi. Movement and activity consistently reduced from 4 to 7 dpi until death. This phenomenon is likely connected with the decreasing trend of body temperature; thus, we perceived that the change in body temperature of the mice was the key indicator of survival. With such a drastic change of body temperature, the mice became moribund, and lung fibrosis did not progress. Judging from the high lethality, we speculated that K18-hACE2 mice have extreme sensitivity to SARS-CoV-2, which made it difficult to analyse the stages of lung disease progression. In line with previous studies showing that the immune response to SARS-CoV-2 in K18-hACE2 mice could mimic that of humans, including the cytokine storm (12, 13), we confirmed high infiltration of immune cells (PTPRC^+^, CD3^+^, neutrophil, and F4/80^+^ cells) in the lungs, which increased in a time-dependent manner.

By contrast, SARS-CoV-2-infected SFTPB-hACE2 and SCGB1A1-hACE2 mice showed distinct lung lesions and immune cell distributions from those of K18-hACE2 mice. Besides having different viral target cells, the immune cell infiltration pattern, primary damaged site and area, and pattern of damage progression varied among the mouse lines. Since pulmonary surfactants, including SFTPB, are synthesised and activated in type II alveolar cells in the lungs (20–22), the hACE2 expression area and primary SARS-CoV-2-infected site was the alveolar region in SFTPB-hACE2 mice. Inflammation, immune cell infiltration near the bronchiole, and capillary dilatation of the lungs increased at the early point of infection (1–2 dpi), and then the damaged area decreased at 5 and 7 dpi. However, the overall severity of lung damage was weaker than that in K18-hACE2 mice. We assumed that the difference in immune cell distributions in each mice model might be closely related to the severity of infection and viral clearance rates. The data that we found is not sufficient to explain this complicated immune cell kinetics. However, the brief increase in the number of F4/80 positive macrophages, one of the innate immune cell populations, at 2dpi and decrease at 5, 7dpi in SFTPB-hACE2 mice could be explained by the fact that SFPTB-hACE2 mice showed lowest viral titer and total viral clearance at 7dpi. On the other hand in SCGB1A1-hACE2 mice, the innate immune cells including F4/80 positive macrophages alone could not contain the level of virus infection at 2dpi, which led the increased number of PTPRC and CD3 positive adaptive immune cells at 5, 7dpi. We expect that the number of immune cells will decrease when viral clearance is completed as SFTPB-hACE2 mice did. K18-hACE2 mice showed drastic increase in the number of innate immune cells, especially F4/80 positive macrophages at 5, 7dpi. However, unlike the other two mice models the severity of SARS-CoV-2 infection was too high which led to the increased number of neutrophils in blood and systemic cytokine storm as reported in previous studies. With respect to SCGB1A1-hACE2 mice, the lung pathology score also increased in a time-dependent manner; however, the damage was much less severe than that in K18-hACE2 mice. The immune cell infiltration in the peripheral blood vessels and oedema of the lungs increased at 1 dpi and then gradually increased until 7 dpi.

We supposed that the different promoters of these three hACE2 transgenic mouse models, *K18*, *SFTPB*, and *Scgb1a1*, were the main contributors to this discrepancy of lung lesions caused by SARS-CoV-2 infection. SARS-CoV-2 targets type I pneumocytes and epithelial cells in various organs in K18-hACE2 mice (12–14, 16). Therefore, severe lesions form proximal to the distal region of the lung, and other organs, including the small intestine, spleen, and brain, were also affected by SARS-CoV-2. Because the *SFTPB* promoter is specifically expressed in type II pneumocytes (20–22), SARS-CoV-2 only infects the distal lung region of SFTPB-hACE2 mice, and thus the pathology score was based on only this focal infection. The *Scgb1a1* promoter is expressed in Clara cells, ciliated cells, goblet cells, and basal cells in the airway epithelium (23–25), and SCGB1A1-expressing basal cells have ability to differentiate to pneumocytes (25, 42). Therefore, SARS-CoV-2 affects not only the bronchos but also a large part of the lung, including the alveolar septa (upper and lower bronchos) and the distal lung septa in SCGB1A1-hACE2 mice.

SARS-CoV-2 has been reported to cause pathological lesions in various organs of K18-hACE2 mice, resulting in respiratory system pathology, including pneumonia in the lung; spleen lesions; gastrointestinal disorder symptoms, especially in the small intestine; and brain damage (36, 38, 43, 44). In the spleen, necrosis of the white pulp and the number of spleen lesions increased, and the white pulp area decreased during the course of SARS-CoV-2 infection in K18-hACE2 mice, along with changes in immune cell dynamics.

However, SFTPB-hACE2 and SCGB1A1-hACE2 mice did not exhibit goblet cell hyperplasia, villi atrophy, and necrosis of the villi lesions in the small intestine following SARS-CoV-2 infection. This difference from the effects in K18-hACE2 mice might be related to functional loss of digestion and absorption of the small intestine, which we speculate is linked to the body weight loss in K18-hACE2 mice. As mentioned above, SARS-CoV-2 spread throughout the body of K18-hACE2 mice causing the observed intestinal disorder. The CBC data showed that neutrophils increased in the peripheral blood of SARS-CoV-2-infected K18-hACE2 mice in a time-dependent manner, and monocytes significantly increased at 5 and 7 dpi compared with those of the non-infected control group. This result further supports that SARS-CoV-2 infects the entire body of K18-hACE2 mice; indeed, the small intestine, spleen, and brain were all damaged by SARS-CoV-2 in this model.

Brain damage was evident by lesions with perivesicular coffing in SARS-CoV-2-infected K18-hACE2 mice. Other studies have indicated that SARS-CoV-2-related mouse morbidity was correlated with neuro-invasion of the virus. Although the precise infection route of SARS-CoV-2 to the brain remains unclear, it has been suggested that the S protein first passes through the blood-brain-barrier, followed by direct infection of central nervous system cells, which have not yet been identified, and then the virus finally travels through the brain *via* the olfactory bulb (10, 14, 17, 37, 45). We detected SARS-CoV-2 in infected K18-hACE2 mice as of 5 dpi, suggesting that the virus spread to the brain *via* whole-body infection through an unknown pathway rather than *via* direct infection of the brain. However, further studies are needed to verify this hypothesis.

The use of lung-specific promoters in the development of SFTPB-hACE2 and SCGB1A1-hACE2 mice resulted in no lesions in the spleen, small intestine, and brain. Thus, these newly developed mouse lines show good potential as suitable lung-specific models of infections with respiratory viruses. We anticipate that the pathophysiological data and mouse lines developed in this study will help to expand research and progress in understanding the pathology of SARS-CoV-2 and other respiratory viruses.

## Materials and methods

### Animals

K18-hACE2 mice [Tg(K18-ACE2)2 Prlmn/J], established in the C57BL/6 background, were purchased from Jackson Laboratory (Bar Harbor, ME, USA). SCGB1A1-hACE2 and SFTPB-hACE2 transgenic mice were newly established in this study from FVB/NJ mice. Human *ACE2* cDNA (HG10108-UT; Sino Biological, Inc., Chesterbrook, PA, USA) was cloned behind the murine *Scgb1a1* (MSCV Puro-CCSP : GFP, #67487, Addgene, Watertown, MA, USA) or human *SFTPB* promoter (#88868, Addgene), generating lung-specific hACE2 expression vectors. Linearized and purified DNA fragments were injected into pronuclei of mouse embryos at 1 ng/μL in 10 mM Tris-HCl and 0.1 mM EDTA (pH 7.5) or 40 ng/μL in 10 mM Tris-HCl and 0.25 mM EDTA (pH 7.4).

Founder mice (pups) were identified by PCR with the primers *Scgb1a1* forward 5′- GAGAATGTCCAAAACATGAA-3′, *Scgb1a1* reverse 5′-AGACCCATTTTGCTGAAGAG-3′; *SFTPB* forward 5′-CTTGTCTCTGACTCAGGGTATTT-3′ and *SFTPB* reverse 5′- CAACCGTTTGCTCTTGTCTTC-3′.

Animal experimental protocols were approved by the Institutional Animal Care and Use Committee (IACUC: 2020-0216, BA-2008-301-071-03) at Yonsei University College of Medicine and were executed in an Association for Assessment and Accreditation of Laboratory Animal Care (AALAC)-accredited unit (#001071).

### Virus

For virus production, we used the Vero African green monkey kidney cell line (Korean Cell Line Bank #10081). Vero cells were maintained at 37 °C in a 5% CO_2_ humidified incubator within DMEM supplemented with 2 mM l-glutamine, 100 units/mL penicillin, 100 μg/mL streptomycin, and 5% FBS. SARS-CoV-2 was obtained from the National Culture Collection for pathogens of Osong, Korea (NCCP 43326, S type), which was isolated from a Korean COVID-19 patient.

Viral culture and titre determination were performed by the plaque assay as described previously (46). In brief, serially diluted supernatants of lung homogenates were added to Vero cells in 6-well plates and incubated at 37 °C for 1 h with gentle agitation every 15 min. The cells were overlaid with DMEM and 1% SeaPlaque agarose (Lonza), 2% FBS, 100 units/mL penicillin, and 100 μg/mL streptomycin. After incubation for 72 h, the cells were fixed with 4% paraformaldehyde and stained with 0.5% crystal violet/20% methanol (v/v). The virus titre was quantified as PFU/g of tissue.

### Infection of mice with SARS-CoV-2

Nine-week-old mice were anaesthetised with a 30 mg/kg of Zoletil and 10 mg/mL of Rompun mixture and 10^5^ PFU SARS-CoV-2 was infected intranasally to K18-hACE2, SFTPB-hACE2, and SCGB1A1-hACE2 transgenic mice; for mock infection, we used an equal volume of PBS. The health, body weight, and body temperature of the mice were monitored daily. Body temperature was measured using an implantable programmable temperature transponder. All animal experiments with SARS-CoV-2 were conducted in a biosafety level-3 facility in accordance with safety guidelines.

### Histological analysis and immunohistochemistry

The mice were euthanised by CO_2_ and fixed with 10% neutral buffered formalin (Sigma, St. Louis, MO, USA) for 24 h. All tissues (lungs, spleen, brain, small intestine) were embedded in paraffin wax, routinely processed, and sectioned at 4 μm thickness for H&E staining and IHC. For H&E staining, de-paraffinized slides were dipped into 0.1% Mayer’s haematoxylin for 10 min and then into 0.5% eosin. The stained slides were dehydrated in an ascending serial grade of 50%, 70%, 95%, and 100% ethanol, and mounted with mount solution (Thermo Fisher Scientific Inc., Waltham, MA, USA). Stained slides were analysed by animal pathologists.

For IHC, the slides were passed through xylene; 100%, 95%, and 70% ethanol; and distilled water for de-paraffinization and rehydration. To perform the antigen retrieval step, pH 6.0 citrate buffer (Dako S1699; Agilent Technologies, Santa Clara, CA, USA) was used under high temperature in a high-pressure cooker and then cooled on ice for 1 h. The slides were treated with 3% H_2_O_2_ in PBS for 30 min to block endogenous peroxidase activity, followed by treatment with M.O.M reagent (Vector Laboratories, Burlingame, CA, USA) for 1 h, and incubated with the following mouse primary antibodies at 4 °C overnight: SARS-CoV-2 N protein (NB100-56576, Novus and 40143-MM08, Sino Biological), SCGB1A1 (ABS1673, Sigma), LAMP3 (DDX0191P, Novus), AGER (MAB1179, R&D Systems), CD3b (ab5690, Abcam), PTPRC (ab64100, Abcam), F4/80 (ab6640, Abcam), Ly-6G/Ly-6C (ab2557, Abcam), doublecortin (SC-271390, Santa Cruz), CD68 (ab31630), nNOS (ab1376), Neu N (ab104224), GFAP (SC-33673).

The slides were treated with a protein blocking solution (DAKO) for 1 h and then incubated with HRP-conjugated secondary antibody (DAKO) for 15 min. DAB substrate (DAKO) was used for development of the IHC signal, and slides were dipped into Mayer’s haematoxylin for nuclear staining. Alexa 488-conjugated anti-mouse, rat, and goat IgG, and Cy3-conjugated anti-rabbit IgG were used for immunofluorescence staining, and images were obtained on a Zeiss LSM980 confocal microscope.

### *In situ* hybridisation

We performed hACE2 and SARS-CoV-2 *in situ* hybridisation using the RNAscope kit (ACD) according to the manufacturer’s protocol. In brief, for deparaffinization and dehydration, the paraffin slides were passed through xylene and a 100% and 95% ethanol gradient twice. After air drying, the slides were treated with hydrogen peroxide, target retrieval solution, and protease K to enclose RNA. For amplification of the RNA signal, the slides were treated with amplifying reagent (ACD) for 2 h and the signal was detected with Fast Red reagent (ACD).

### Transmission electron microscopy

The lung, spleen, small intestine, and brain specimens were pre-fixed in 2% glutaraldehyde/paraformaldehyde in 0.1 M PBS and washed in 0.1 M phosphate buffer for 24 h at 4 °C for TEM. Post-fixation was performed with 1% OsO_4_ in 0.1 M phosphate buffer for 2 h, followed by dehydration in an ascending serial grade of ethanol (50%, 60%, 70%, 80%, 90%, 95%, 100%) for 10 min each, and incubated with propylene oxide for 10 min. The specimens were embedded in Poly/Bed 812 kit (Polyscience) and the blocks were cut into 200-nm sections with an ultramicrotome. The sections were placed on a copper grid and imaged with TEM (JEM-1011, JEOL, Tokyo, Japan) at an acceleration voltage of 80 kV equipped with a Mega view III CCD camera (Soft Imaging System, Germany).

### Haematological analysis

Peripheral blood samples were collected from the heart immediately following mouse euthanasia and complete blood count (CBC) analysis was performed using the BC-5000Vet system (Shenzhen Mindray Bio-Medical Electronics Co., China).

### Statistical analysis

Statistical significance was calculated using PRISM v9.0 software (GraphPad Software, San Diego, CA).

## Data availability statement

The datasets presented in this study can be found in online repositories. The names of the repository/repositories and accession number(s) can be found in the article/**Supplementary Material**.

## Ethics statement

The animal study was reviewed and approved by Institutional Animal Care and Use Committee (IACUC: 2020-0216, BA-2008-301-071-03) at Yonsei University College of Medicine and Association for Assessment and Accreditation of Laboratory Animal Care (AALAC)-accredited unit (#001071).

## Author contributions

Conceptualization and Data curation: SHK, JSK, JYJ, HAN, DKL, HL, KTN, JKS. Formal analysis: SHK, JSK, JYJ, HAN, JSP. Investigation: SHK, JSK, JYJ, HAN, JSP, CYU, HJO, KRC, YWL, HJJ, IHP, JYO, JSS, JJK, JYS. Project administration: SHK, JSK, JYJ, HAN, DIO, SYY, SYL, SPK, SYK, YBK, JYH. Resources: SHK, JSK, JYJ, HAN, YJ, SMH, SHA, KSC. Supervision: SHK, JSK, JYJ, HAN, JWY, JSS, HYL, KMK, DKL, HL, KTN, JKS. Validation: SHK, JSK, JYJ, HAN, HDJ, DHJ, SHS, YJL, HJL, HBK, JWP. Writing and Editing: SHK, JSK, JYJ, HAN, DKL, HL, KTN, JKS. All authors contributed to the article and approved the submitted version.

## Funding

This project was supported by the Korea Mouse Phenotyping Project (NRF-2016M3A9D5A01952416 and 2021M3H9A1030260) and by the Brain Korea 21 Project for Medical Science at Yonsei University. KTN is supported by the Bio and Medical Technology Development Program of the National Research Foundation (NRF) funded by the Korean government (MSIT) (2021M3H9A1038083).

## Conflict of interest

The authors declare that the research was conducted in the absence of any commercial or financial relationships that could be construed as a potential conflict of interest.

## Publisher’s note

All claims expressed in this article are solely those of the authors and do not necessarily represent those of their affiliated organizations, or those of the publisher, the editors and the reviewers. Any product that may be evaluated in this article, or claim that may be made by its manufacturer, is not guaranteed or endorsed by the publisher.

## Supplementary material

Supplementary Figure 1 Novel human ACE2 (hACE2) transgenic mouse models established using *SFTPB* and *Scgb1a1* promoters. **(A)** hACE2 protein expression in various tissue in wild-type and SFTPB-hACE2 mice. **(B)** hACE2 expression in the lung and ileum in SCGB1A1-hACE2 mice. **(C, D)** hACE2 expression in the lung analysed by *in situ* hybridisation in wild-type and hACE2 transgenic mice.

Supplementary Figure 2 Relative tissue weight in SARS-CoV-2-infected animals. Tissue weights were measured during autopsy and normalised to total body weight. Data represent mean ± standard error (K18-hACE2 and SFTPB-hACE2, n = 25; SCGB1A1-hACE2, n = 12) and non-infected control animals (K18-hACE2 and SFTPB-hACE2, n = 5; SCGB1A1-hACE2, n = 3). (*P* < 0.05; ** *P* < 0.01).

Supplementary Figure 3 Histopathological analysis of the SARS-CoV-2-infected lung in K18-hACE2, SFTPB-hACE2, and SCGB1A1-hACE2 mice. **(A–C)** H&E staining of the lung following intranasally infection of 1 × 10^5^ PFU SARS-CoV-2 in K18-hACE2 **(A)**, SFTPB-hACE2 **(B)**, and SCGB1A1-hACE2 **(C)** mice. Autopsy was conducted at 1, 2, 5, and 7 dpi. The scale bars are 100 μm (left panels) and 50 μm (right panels).

Supplementary Figure 4 Immune cell infiltration and distribution in the SARS-CoV-2-infected lungs of K18-hACE2, SFTPB-hACE2, and SCGB1A1-hACE2 mice. PTPRC^+^ cells **(A)**, CD3^+^ cells **(B)**, neutrophil **(C)**, and F4/80^+^ macrophage **(D)** distribution in the lungs confirmed by immunohistochemistry. Each marker is stained brown and counterstained by DAPI (blue).

Supplementary Figure 5 Immunohistochemistry analysis of SARS-CoV-2 infectious target lung cells in K18-hACE2, SFTPB-hACE2, and SCGB1A1-hACE2 mice. N protein of SARS-CoV-2 (red) and lung cell type markers are double stained: **(A)** type I alveolar cell marker AGER, **(B)** type II alveolar cell marker LAMP3, **(C)** club cell marker SCGB1A1, **(D)** ciliated cell marker acetylated alpha-tubulin, **(E)** pulmonary neuroendocrine cell marker chromogranin A, and **(F)** epithelial goblet cell marker MUC5AC.

Supplementary Figure 6 SARS-CoV-2 particles detected in the lungs of K18-hACE2 and SCGB1A1-hACE2 mice. SARS-CoV-2 particles detected by electron microscopy. In K18-hACE2 mice, viral particles (black arrow heads) were distributed in type I alveolar pneumocytes **(A)**. The dotted line is the type II alveolar pneumocyte. M, mitochondria; LB, lamellar body. In SCGB1A1-hACE2 mice, SARS-CoV-2 is bound to the cilia in bronchos **(B)**.

Supplementary Figure 7 Histopathological analysis of the spleen in SARS-CoV-2-infected K18-hACE2, SFTPB-hACE2, and SCGB1A1-hACE2 mice. **(A–C)** Pathological analysis of the spleen following intranasal infection of 1 × 10^5^ PFU SARS-CoV-2 in K18-hACE2 **(A)**, SFTPB-hACE2 **(B)**, and SCGB1A1-hACE2 **(C)** mice. Autopsy was conducted at 1, 2, 5, and 7 dpi. The scale bars are 100 μm (left panels) and 50 μm (right panels).

Supplementary Figure 8 Immune cell distribution in the SARS-CoV-2-infected spleen of K18-hACE2 mice. The B cell marker PTPRC, T cell marker CD3, and macrophage marker F4/80 were stained in SARS-CoV-2-infected K18-hACE2 mice.

Supplementary Figure 9 Lesion and infectious target cells in the brain of SARS-CoV-2-infected SFTPB-hACE2 and SCGB1A1-hACE2 mice. Brain lesion, diagnosed as perivesicular coffing, was not detected in the brains of SFTPB-hACE2, SCGB1A1-hACE2, and control mice at 5 dpi **(A)**. SARS-CoV-2 infectious target cells in the brain of K18-hACE2 mice **(B)**. SARS-CoV-2 viral particles were detected in K18-hACE2 mice (red arrow) **(C)**.

## References

[1] Wu F Zhao S Yu B Chen YM Wang W Song ZG. A new coronavirus associated with human respiratory disease in China. Nature. (2020) 579(7798):265–9. doi: 10.1038/s41586-020-2202-3 PMC7094943 32015508

[2] Coronaviridae Study Group of the International Committee on Taxonomy of V. The species severe acute respiratory syndrome-related coronavirus: classifying 2019-nCoV and naming it SARS-CoV-2. Nat Microbiol (2020) 5(4):536–44. doi: 10.1038/s41564-020-0695-z PMC7095448 32123347

[3] Cherian S Potdar V Jadhav S Yadav P Gupta N Das M. SARS-CoV-2 spike mutations, L452R, T478K, E484Q and P681R, in the second wave of COVID-19 in maharashtra, India. Microorganisms. (2021) 9(7):1542. doi: 10.3390/microorganisms9071542 34361977 PMC8307577

[4] Abu-Raddad LJ Chemaitelly H Butt AA National Study Group for C-V. Effectiveness of the BNT162b2 covid-19 vaccine against the B.1.1.7 and B.1.351 variants. N Engl J Med (2021) 385(2):187–9. doi: 10.1056/NEJMc2104974 PMC8117967 33951357

[5] Hoffmann M Kleine-Weber H Schroeder S Kruger N Herrler T Erichsen S. SARS-CoV-2 cell entry depends on ACE2 and TMPRSS2 and is blocked by a clinically proven protease inhibitor. Cell. (2020) 181(2):271–80.e8. doi: 10.1016/j.cell.2020.02.052 32142651 PMC7102627

[6] Ziegler CGK Allon SJ Nyquist SK Mbano IM Miao VN Tzouanas CN. SARS-CoV-2 receptor ACE2 is an interferon-stimulated gene in human airway epithelial cells and is detected in specific cell subsets across tissues. Cell. (2020) 181(5):1016–35.e19. doi: 10.1016/j.cell.2020.04.035 32413319 PMC7252096

[7] Sungnak W Huang N Becavin C Berg M Queen R Litvinukova M. SARS-CoV-2 entry factors are highly expressed in nasal epithelial cells together with innate immune genes. Nat Med (2020) 26(5):681–7. doi: 10.1038/s41591-020-0868-6 PMC8637938 32327758

[8] McCray PB Jr. Pewe L Wohlford-Lenane C Hickey M Manzel L Shi L. Lethal infection of K18-hACE2 mice infected with severe acute respiratory syndrome coronavirus. J Virol (2007) 81(2):813–21. doi: 10.1128/JVI.02012-06 PMC1797474 17079315

[9] Jia H Yue X Lazartigues E. ACE2 mouse models: a toolbox for cardiovascular and pulmonary research. Nat Commun (2020) 11(1):5165. doi: 10.1038/s41467-020-18880-0 33057007 PMC7560817

[10] Moreau GB Burgess SL Sturek JM Donlan AN Petri WA Mann BJ. Evaluation of K18-hACE2 mice as a model of SARS-CoV-2 infection. Am J Trop Med Hyg (2020) 103(3):1215–9. doi: 10.4269/ajtmh.20-0762 PMC7470527 32723427

[11] Rathnasinghe R Strohmeier S Amanat F Gillespie VL Krammer F Garcia-Sastre A. Comparison of transgenic and adenovirus hACE2 mouse models for SARS-CoV-2 infection. Emerg Microbes Infect (2020) 9(1):2433–45. doi: 10.1080/22221751.2020.1838955 PMC7655046 33073694

[12] Oladunni FS Park JG Pino PA Gonzalez O Akhter A Allue-Guardia A. Lethality of SARS-CoV-2 infection in K18 human angiotensin-converting enzyme 2 transgenic mice. Nat Commun (2020) 11(1):6122. doi: 10.1038/s41467-020-19891-7 33257679 PMC7705712

[13] Winkler ES Bailey AL Kafai NM Nair S McCune BT Yu J. SARS-CoV-2 infection of human ACE2-transgenic mice causes severe lung inflammation and impaired function. Nat Immunol (2020) 21(11):1327–35. doi: 10.1038/s41590-020-0778-2 PMC7578095 32839612

[14] Kumari P Rothan HA Natekar JP Stone S Pathak H Strate PG. Neuroinvasion and encephalitis following intranasal inoculation of SARS-CoV-2 in K18-hACE2 mice. Viruses (2021) 13(1):132. doi: 10.3390/v13010132 33477869 PMC7832889

[15] Gan ES Syenina A Linster M Ng B Zhang SL Watanabe S. A mouse model of lethal respiratory dysfunction for SARS-CoV-2 infection. Antiviral Res (2021) 193:105138. doi: 10.1016/j.antiviral.2021.105138 34246735 PMC8264561

[16] Yinda CK Port JR Bushmaker T Offei Owusu I Purushotham JN Avanzato VA. K18-hACE2 mice develop respiratory disease resembling severe COVID-19. PLoS Pathog (2021) 17(1):e1009195. doi: 10.1371/journal.ppat.1009195 33465158 PMC7875348

[17] Golden JW Cline CR Zeng X Garrison AR Carey BD Mucker EM. Human angiotensin-converting enzyme 2 transgenic mice infected with SARS-CoV-2 develop severe and fatal respiratory disease. JCI Insight (2020) 5(19):e142032. doi: 10.1172/jci.insight.142032 32841215 PMC7566707

[18] Carossino M Kenney D O'Connell AK Montanaro P Tseng AE Gertje HP. Fatal neurodissemination and SARS-CoV-2 tropism in K18-hACE2 mice is only partially dependent on hACE2 expression. Viruses (2022) 14(3):535. doi: 10.3390/v14030535 35336942 PMC8955233

[19] Dong W Mead H Tian L Park JG Garcia JI Jaramillo S. The K18-human ACE2 transgenic mouse model recapitulates non-severe and severe COVID-19 in response to an infectious dose of the SARS-CoV-2 virus. J Virol (2022) 96(1):e0096421. doi: 10.1128/JVI.00964-21 34668775 PMC8754221

[20] Sin DD Tammemagi CM Lam S Barnett MJ Duan X Tam A. Pro-surfactant protein b as a biomarker for lung cancer prediction. J Clin Oncol (2013) 31(36):4536–43. doi: 10.1200/JCO.2013.50.6105 PMC3871515 24248694

[21] Liley HG Ertsey R Gonzales LW Odom MW Hawgood S Dobbs LG. Synthesis of surfactant components by cultured type II cells from human lung. Biochim Biophys Acta (1988) 961(1):86–95. doi: 10.1016/0005-2760(88)90133-6 3382694

[22] Lin Z Thorenoor N Wu R DiAngelo SL Ye M Thomas NJ. Genetic association of pulmonary surfactant protein genes, SFTPA1, SFTPA2, SFTPB, SFTPC, and SFTPD with cystic fibrosis. Front Immunol (2018) 9:2256. doi: 10.3389/fimmu.2018.02256 30333828 PMC6175982

[23] Zheng D Limmon GV Yin L Leung NH Yu H Chow VT. Regeneration of alveolar type I and II cells from Scgb1a1-expressing cells following severe pulmonary damage induced by bleomycin and influenza. PLoS One (2012) 7(10):e48451. doi: 10.1371/journal.pone.0048451 23119022 PMC3485240

[24] Wang SZ Rosenberger CL Bao YX Stark JM Harrod KS. Clara Cell secretory protein modulates lung inflammatory and immune responses to respiratory syncytial virus infection. J Immunol (2003) 171(2):1051–60. doi: 10.4049/jimmunol.171.2.1051 12847279

[25] Rawlins EL Okubo T Xue Y Brass DM Auten RL Hasegawa H. The role of Scgb1a1+ Clara cells in the long-term maintenance and repair of lung airway, but not alveolar, epithelium. Cell Stem Cell (2009) 4(6):525–34. doi: 10.1016/j.stem.2009.04.002 PMC2730729 19497281

[26] Chevrier S Zurbuchen Y Cervia C Adamo S Raeber ME de Souza N. A distinct innate immune signature marks progression from mild to severe COVID-19. Cell Rep Med (2021) 2(1):100166. doi: 10.1016/j.xcrm.2020.100166 33521697 PMC7817872

[27] Lucas C Wong P Klein J Castro TBR Silva J Sundaram M. Longitudinal analyses reveal immunological misfiring in severe COVID-19. Nature. (2020) 584(7821):463–9. doi: 10.1038/s41586-020-2588-y PMC7477538 32717743

[28] Veras FP Pontelli MC Silva CM Toller-Kawahisa JE de Lima M Nascimento DC. SARS-CoV-2-triggered neutrophil extracellular traps mediate COVID-19 pathology. J Exp Med (2020) 217(12):e20201129. doi: 10.1084/jem.20201129 32926098 PMC7488868

[29] Hazeldine J Lord JM. Neutrophils and COVID-19: Active participants and rational therapeutic targets. Front Immunol (2021) 12:680134. doi: 10.3389/fimmu.2021.680134 34149717 PMC8206563

[30] Fraga-Silva TFC Maruyama SR Sorgi CA Russo EMS Fernandes APM de Barros Cardoso CR. COVID-19: Integrating the complexity of systemic and pulmonary immunopathology to identify biomarkers for different outcomes. Front Immunol (2020) 11:599736. doi: 10.3389/fimmu.2020.599736 33584667 PMC7878380

[31] Antonioli L Fornai M Pellegrini C Blandizzi C. NKG2A and COVID-19: another brick in the wall. Cell Mol Immunol (2020) 17(6):672–4. doi: 10.1038/s41423-020-0450-7 PMC7203720 32382127

[32] Gelzo M Cacciapuoti S Pinchera B De Rosa A Cernera G Scialo F. Prognostic role of neutrophil to lymphocyte ratio in COVID-19 patients: Still valid in patients that had started therapy? Front Public Health (2021) 9:664108. doi: 10.3389/fpubh.2021.664108 34211953 PMC8239130

[33] Zahorec R Hulin I Zahorec P. Rationale use of neutrophil-to-lymphocyte ratio for early diagnosis and stratification of COVID-19. Bratisl Lek Listy. (2020) 121(7):466–70. doi: 10.4149/BLL_2020_077 32989997

[34] Laforge M Elbim C Frere C Hemadi M Massaad C Nuss P. Tissue damage from neutrophil-induced oxidative stress in COVID-19. Nat Rev Immunol (2020) 20(9):515–6. doi: 10.1038/s41577-020-0407-1 PMC7388427 32728221

[35] Kuri-Cervantes L Pampena MB Meng W Rosenfeld AM Ittner CAG Weisman AR. Comprehensive mapping of immune perturbations associated with severe COVID-19. Sci Immunol (2020) 5(49):eabd7114. doi: 10.1126/sciimmunol.abd7114 32669287 PMC7402634

[36] Lamers MM Beumer J van der Vaart J Knoops K Puschhof J Breugem TI. SARS-CoV-2 productively infects human gut enterocytes. Science. (2020) 369(6499):50–4. doi: 10.1126/science.abc1669 PMC7199907 32358202

[37] Song E Zhang C Israelow B Lu-Culligan A Prado AV Skriabine S. Neuroinvasion of SARS-CoV-2 in human and mouse brain. J Exp Med (2021) 218(3). doi: 10.1101/2020.06.25.169946 PMC7808299 33433624

[38] Lehmann M Allers K Heldt C Meinhardt J Schmidt F Rodriguez-Sillke Y. Human small intestinal infection by SARS-CoV-2 is characterized by a mucosal infiltration with activated CD8(+) T cells. Mucosal Immunol (2021) 14(6):1381–92. doi: 10.1038/s41385-021-00437-z PMC8379580 34420043

[39] Zhang BZ Chu H Han S Shuai H Deng J Hu YF. SARS-CoV-2 infects human neural progenitor cells and brain organoids. Cell Res (2020) 30(10):928–31. doi: 10.1038/s41422-020-0390-x PMC7399356 32753756

[40] Wuyts WA Agostini C Antoniou KM Bouros D Chambers RC Cottin V. The pathogenesis of pulmonary fibrosis: a moving target. Eur Respir J (2013) 41(5):1207–18. doi: 10.1183/09031936.00073012 23100500

[41] Selman M Pardo A. Idiopathic pulmonary fibrosis: an epithelial/fibroblastic cross-talk disorder. Respir Res (2002) 3:3. doi: 10.1186/rr175 11806838 PMC64814

[42] Ruiz Garcia S Deprez M Lebrigand K Cavard A Paquet A Arguel MJ. Novel dynamics of human mucociliary differentiation revealed by single-cell RNA sequencing of nasal epithelial cultures. Development (2019) 146 (20): dev177428. doi: 10.1242/dev.177428 31558434 PMC6826037

[43] Rizzo R Neri LM Simioni C Bortolotti D Occhionorelli S Zauli G. SARS-CoV-2 nucleocapsid protein and ultrastructural modifications in small bowel of a 4-week-negative COVID-19 patient. Clin Microbiol Infect (2021) 27(6):936–7. doi: 10.1016/j.cmi.2021.01.012 PMC7832059 33465499

[44] Ye Q Wang B Zhang T Xu J Shang S. The mechanism and treatment of gastrointestinal symptoms in patients with COVID-19. Am J Physiol Gastrointest Liver Physiol (2020) 319(2):G245–G52. doi: 10.1152/ajpgi.00148.2020 PMC7414235 32639848

[45] Rhea EM Logsdon AF Hansen KM Williams LM Reed MJ Baumann KK. The S1 protein of SARS-CoV-2 crosses the blood-brain barrier in mice. Nat Neurosci (2021) 24(3):368–78. doi: 10.1038/s41593-020-00771-8 PMC8793077 33328624

[46] Balish AL Katz JM Klimov AI. Influenza: propagation, quantification, and storage. Curr Protoc Microbiol (2013) 29(1):15G.1.1-24. doi: 10.1002/9780471729259.mc15g01s29 23686827

