# Corrigendum: Mouse models of lung-specific SARS-CoV-2 infection with moderate pathological traits

**DOI:** 10.3389/fimmu.2022.1105713

**Published:** 2022-12-02

**Authors:** Sung-Hee Kim, Jiseon Kim, Ji Yun Jang, Hyuna Noh, Jisun Park, Haengdueng Jeong, Donghun Jeon, Chanyang Uhm, Heeju Oh, Kyungrae Cho, Yoon Jeon, Dain On, Suhyeon Yoon, Soo-Yeon Lim, Sol Pin Kim, Youn Woo Lee, Hui Jeong Jang, In Ho Park, Jooyeon Oh, Jung Seon Seo, Jeong Jin Kim, Sang-Hyuk Seok, Yu Jin Lee, Seung-Min Hong, Se-Hee An, Seo Yeon Kim, Young Been Kim, Ji-Yeon Hwang, Hyo-Jung Lee, Hong Bin Kim, Kang-Seuk Choi, Jun Won Park, Jun-Young Seo, Jun-Won Yun, Jeon-Soo Shin, Ho-Young Lee, Kyoungmi Kim, Daekee Lee, Ho Lee, Ki Taek Nam, Je Kyung Seong

**Affiliations:** ^1^ Severance Biomedical Science Institute, Graduate School of Medical Science, Brain Korea 21 Project, Yonsei University College of Medicine, Seoul, South Korea; ^2^ Division of Cancer Biology, Research Institute, National Cancer Center, Goyang, Gyeonggi, South Korea; ^3^ College of Pharmacy, Dongguk University, Seoul, South Korea; ^4^ Korea Mouse Phenotyping Center, Seoul National University, Seoul, South Korea; ^5^ Department of Life Science, Ewha Womans University, Seoul, South Korea; ^6^ Laboratory of Developmental Biology and Genomics, Research Institute for Veterinary Science, and BK21 PLUS Program for Creative Veterinary Science Research, College of Veterinary Medicine, Seoul National University, Seoul, South Korea; ^7^ Department of Nuclear Medicine, Seoul National University Bundang Hospital, Seongnam, South Korea; ^8^ Institute of Immunology and Immunological Diseases, Yonsei University College of Medicine, Seoul, South Korea; ^9^ Department of Microbiology, Yonsei University College of Medicine, Seoul, South Korea; ^10^ Division of Biomedical Convergence, College of Biomedical Science, Kangwon National University, Chuncheon, South Korea; ^11^ Laboratory of Avian Diseases, BK21 plus Program for Veterinary Science and Research Institute for Veterinary Science, College of Veterinary Medicine, Seoul National University, Seoul, South Korea; ^12^ Preclinical Research Center, Seoul National University Bundang Hospital, Seongnam, South Korea; ^13^ Department of Periodontology, Section of Dentistry, Seoul National University Bundang Hospital, Seongnam, South Korea; ^14^ Department of Internal Medicine, Seoul National University Bundang Hospital, Seoul National University College of Medicine, Seongnam, South Korea; ^15^ Laboratory of Veterinary Toxicology, College of Veterinary Medicine, Seoul National University, Seoul, South Korea; ^16^ Department of Nuclear Medicine, College of Medicine, Seoul National University, Seoul, South Korea; ^17^ Department of Physiology and Biomedical Science, Korea University College of Medicine, Seoul, South Korea; ^18^ Graduate School of Cancer Science and Policy, National Cancer Center, Goyang, Gyeonggi, South Korea; ^19^ BIO-MAX Institute, Seoul National University, Seoul, South Korea; ^20^ Interdisciplinary Program for Bioinformatics, Seoul National University, Seoul, South Korea

**Keywords:** SARS-CoV-2, hACE2 transgenic mice, K18-hACE2 mice model, SFTPB-hACE2 mice model, SCGB1A1-hACE2 mice model

In the published article, there was an error in the Funding statement. A grant number was missing for the funder the Korea Mouse Phenotyping Project. The correct Funding statement appears below.

“This project was supported by the Korea Mouse Phenotyping Project (NRF-2016M3A9D5A01952416 and 2021M3H9A1030260) and by the Brain Korea 21 Project for Medical Science at Yonsei University. KTN is supported by the Bio and Medical Technology Development Program of the National Research Foundation (NRF) funded by the Korean government (MSIT) (2021M3H9A1038083).”

The authors apologize for this error and state that this does not change the scientific conclusions of the article in any way. The original article has been updated.

## Publisher’s note

All claims expressed in this article are solely those of the authors and do not necessarily represent those of their affiliated organizations, or those of the publisher, the editors and the reviewers. Any product that may be evaluated in this article, or claim that may be made by its manufacturer, is not guaranteed or endorsed by the publisher.

