# Supplementary figures and images for: Mouse models of lung-specific SARS-CoV-2 infection with moderate pathological traits
Source: Front Immunol. 2022 Nov 15;13:1055811. doi: 10.3389/fimmu.2022.1055811 (PMC9706212; doi:10.3389/fimmu.2022.1055811)

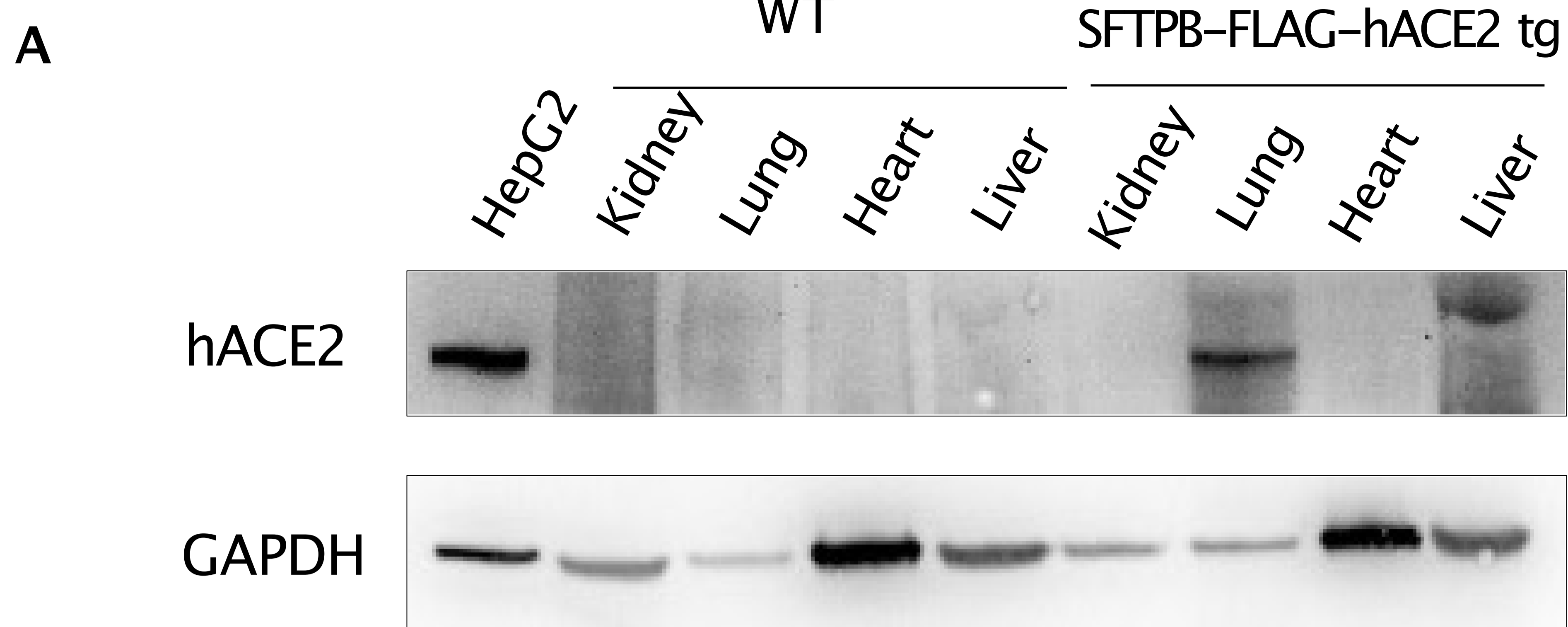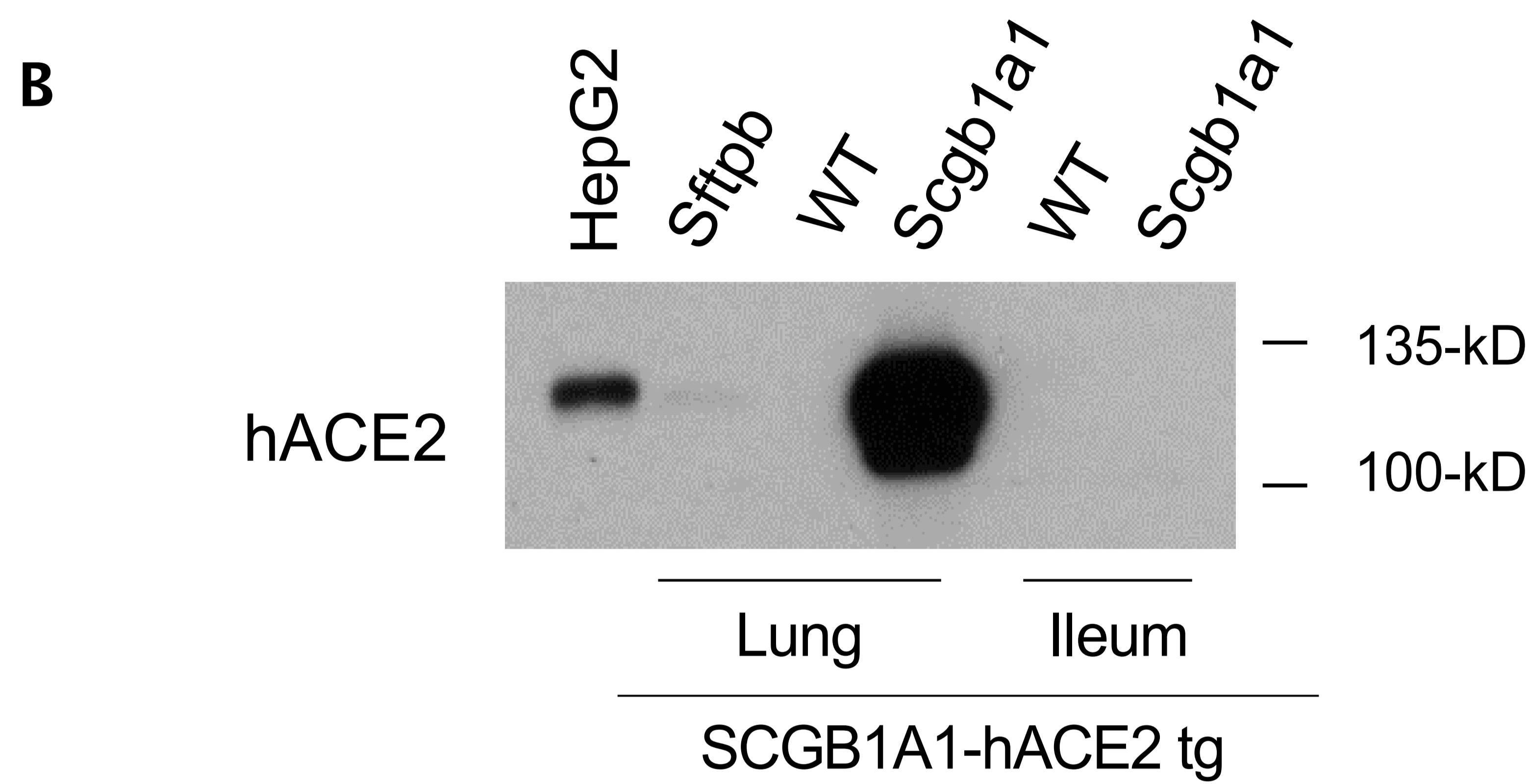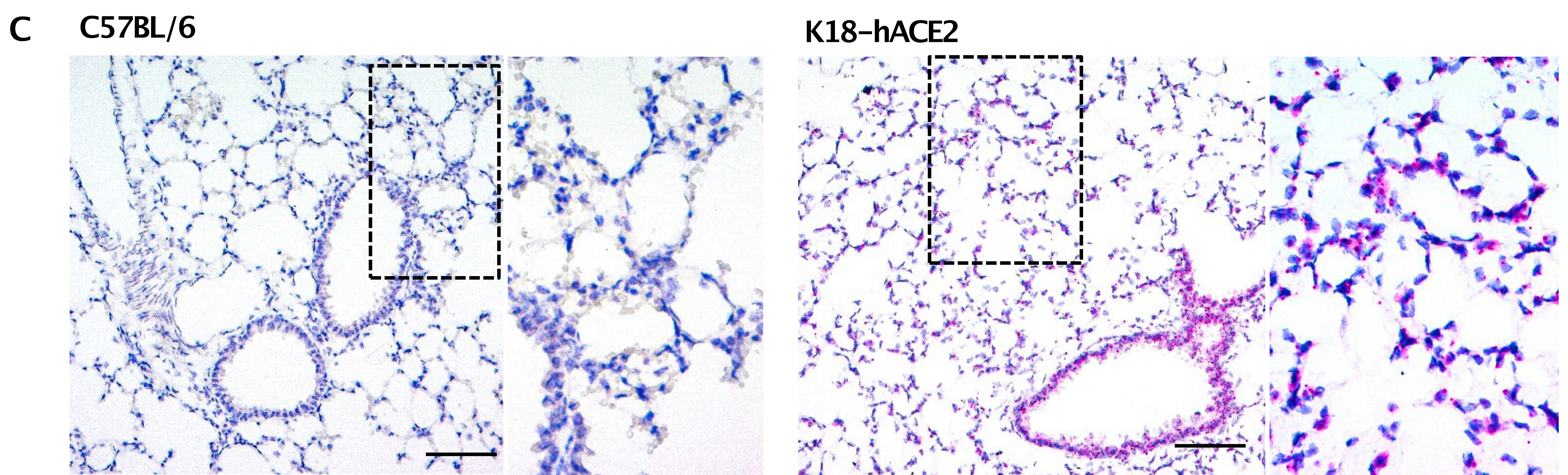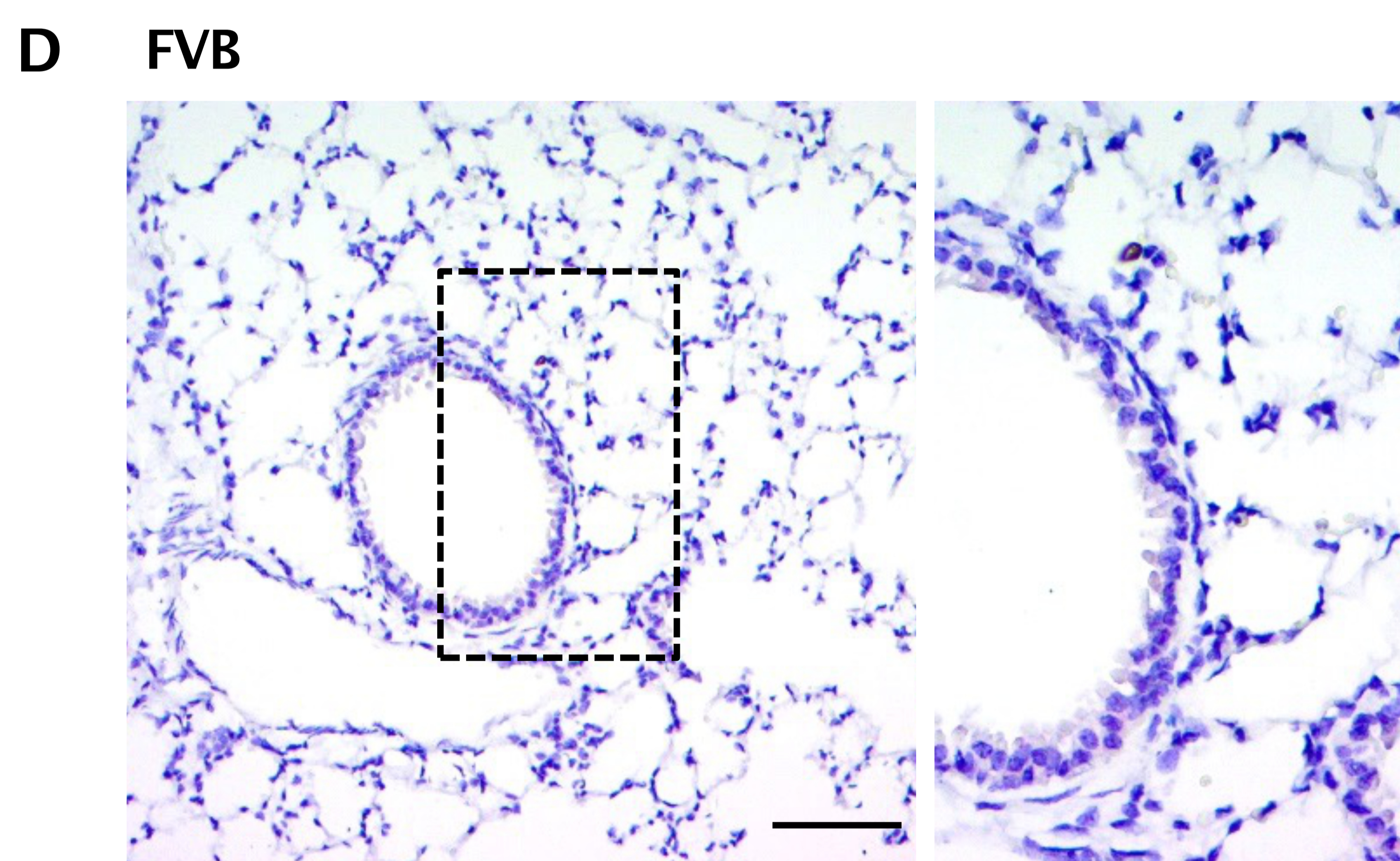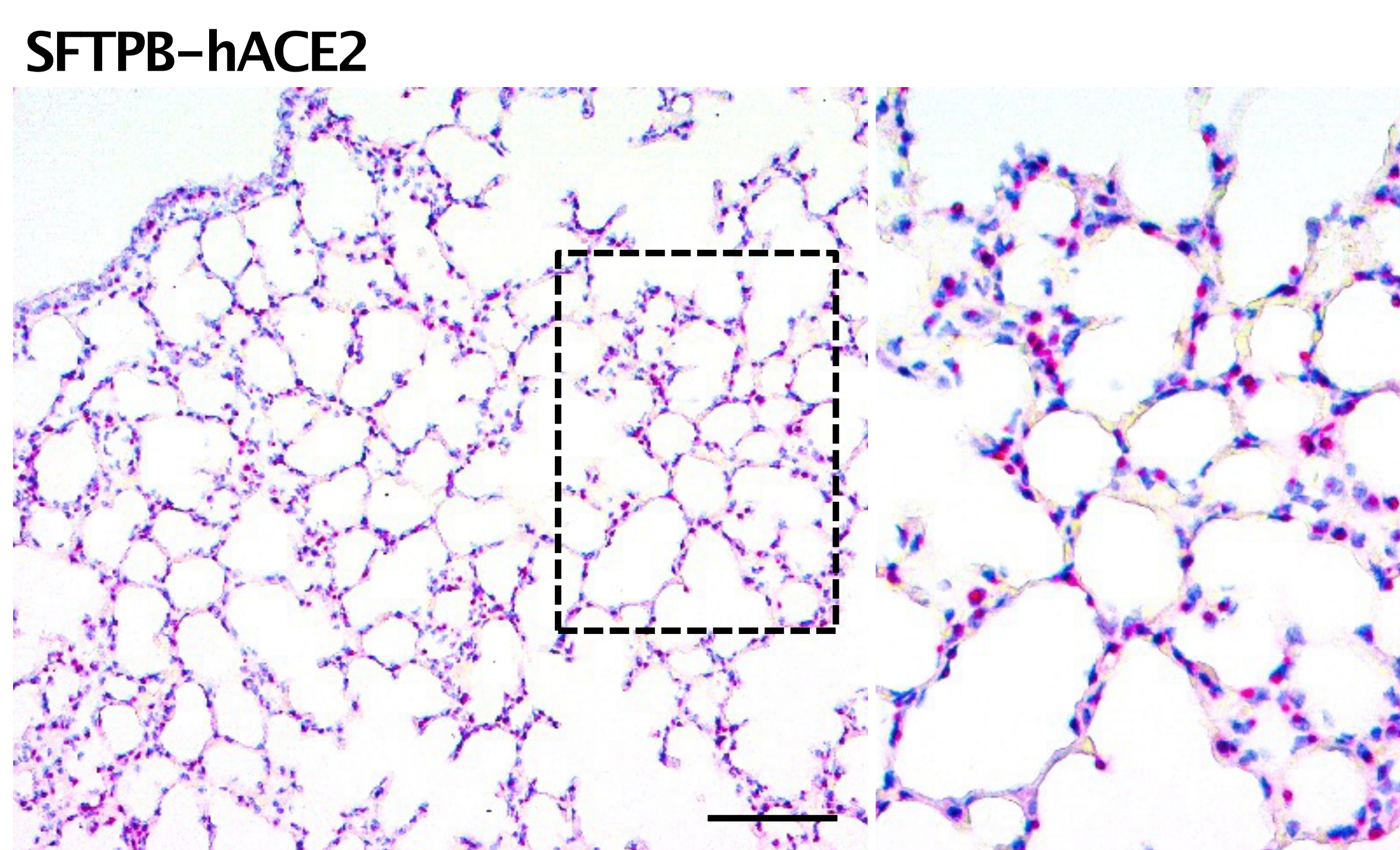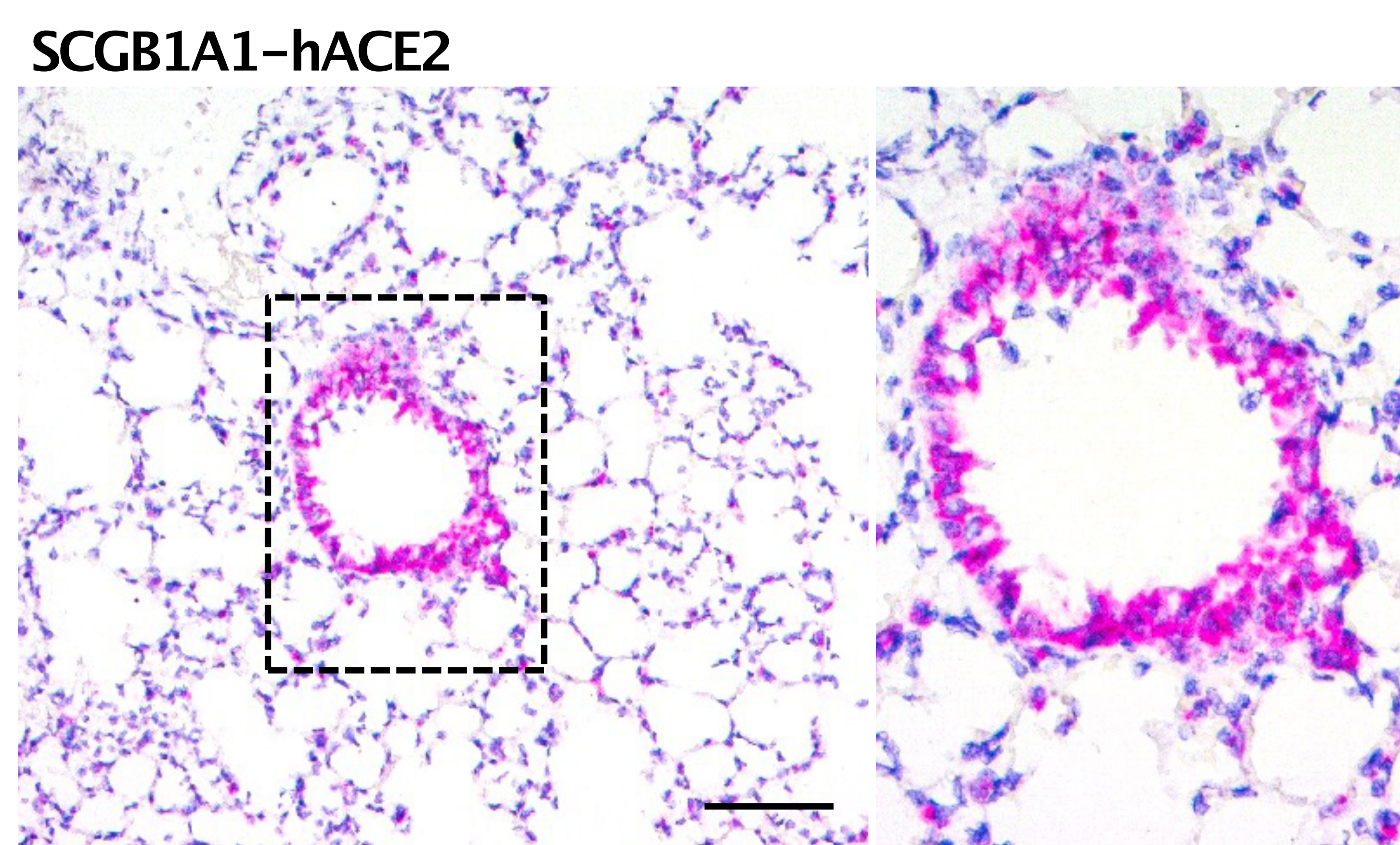

Supplement: Supplementary Figure 1 — Novel human ACE2 (hACE2) transgenic mouse models established using SFTPB and Scgb1a1 promoters. (A) hACE2 protein expression in various tissue in wild-type and SFTPB-hACE2 mice. (B) hACE2 expression in the lung and ileum in SCGB1A1-hACE2 mice. (C, D) hACE2 expression in the lung analysed by in situ hybridisation in wild-type and hACE2 transgenic mice. [file Image_1.pdf]

**A**

# **K18-hACE2**

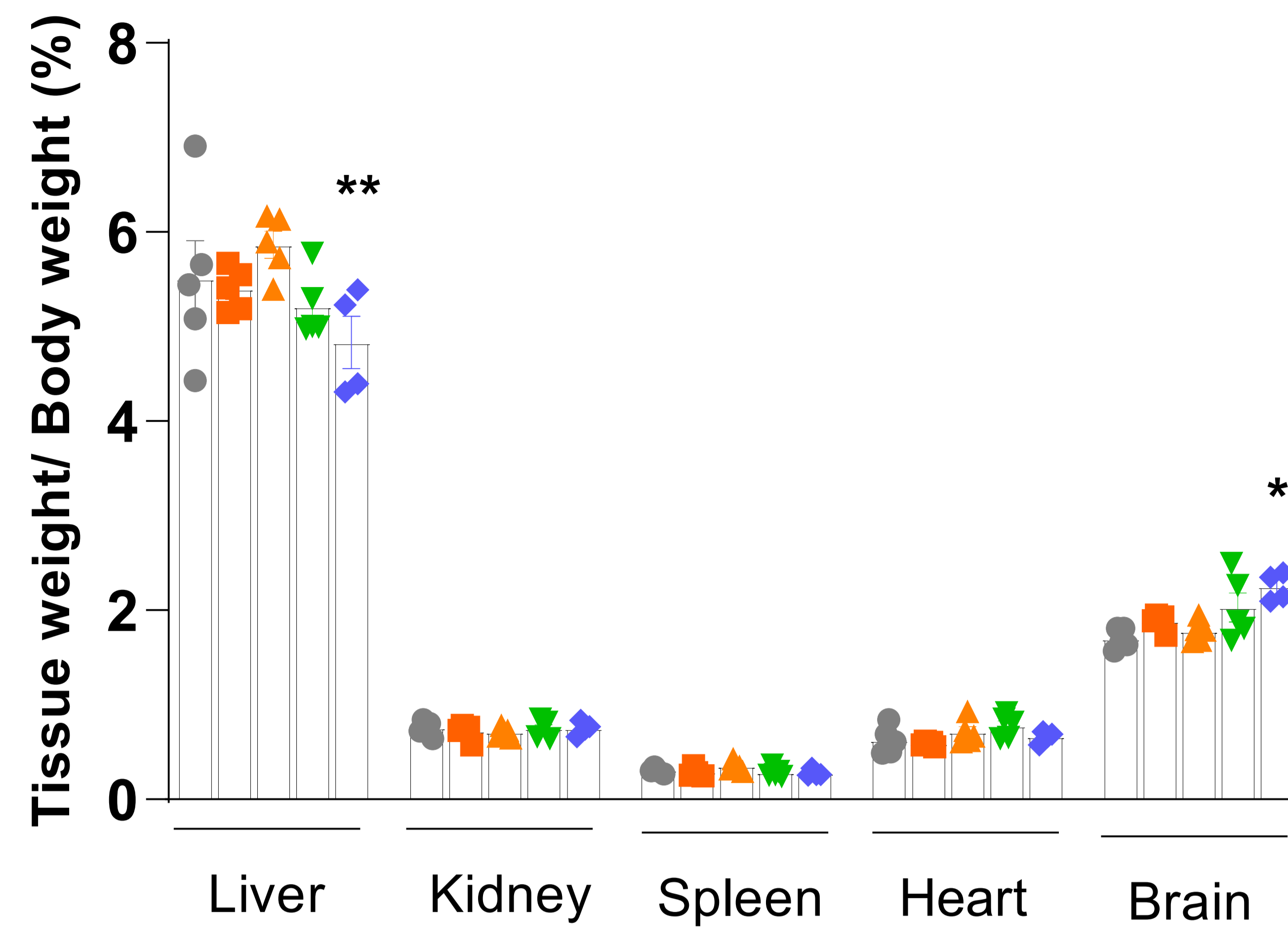

**B**

# **SFTPB-hACE2**

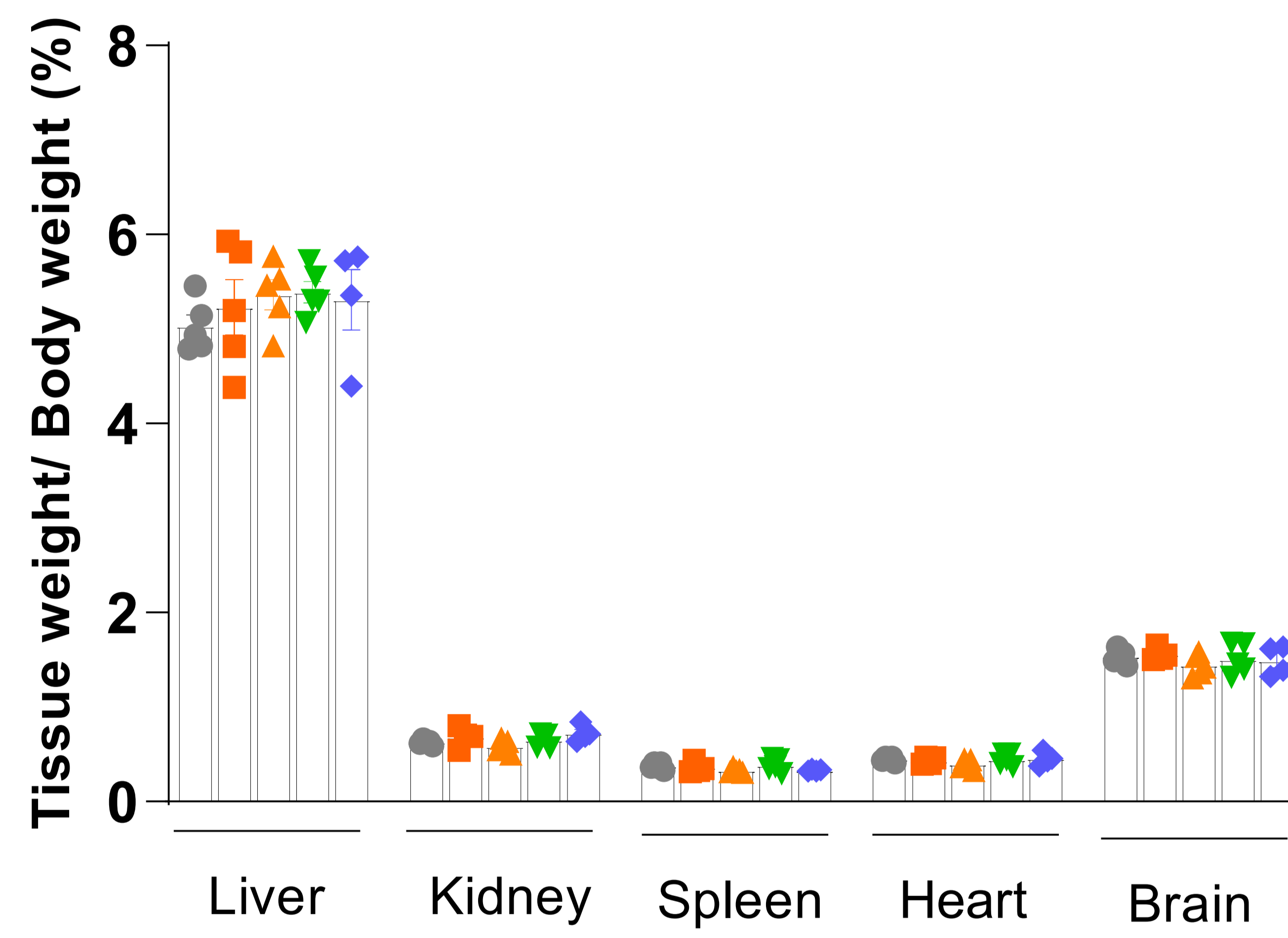

**C**

# **SCGB1A1-hACE2**

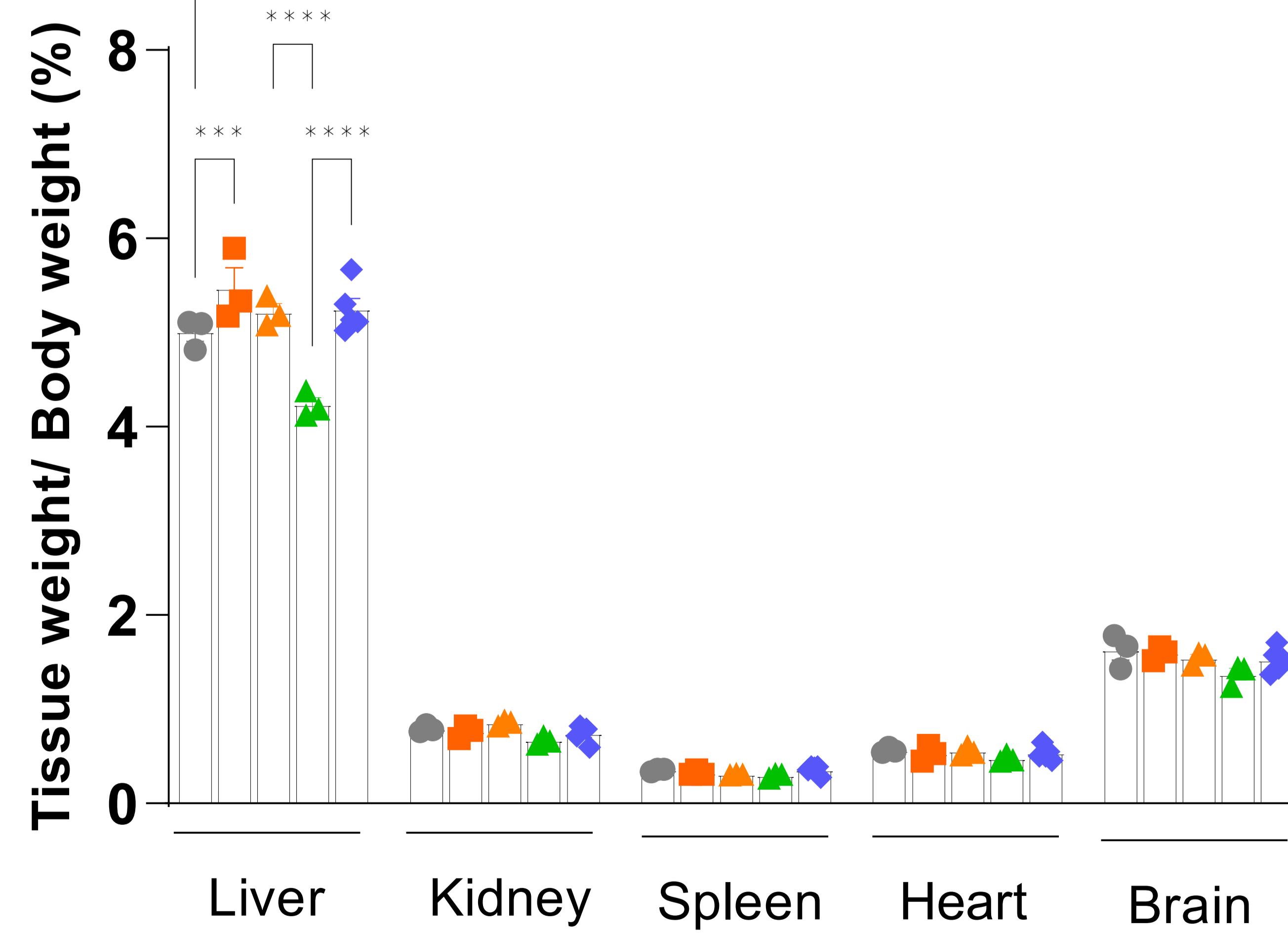

● Control    ■ 1dpi    ▲ 2dpi    ▼ 5dpi    ◆ 7dpi

Supplement: Supplementary Figure 2 — Relative tissue weight in SARS-CoV-2-infected animals. Tissue weights were measured during autopsy and normalised to total body weight. Data represent mean ± standard error (K18-hACE2 and SFTPB-hACE2, n = 25; SCGB1A1-hACE2, n = 12) and non-infected control animals (K18-hACE2 and SFTPB-hACE2, n = 5; SCGB1A1-hACE2, n = 3). (P < 0.05; ** P < 0.01). [file Image_2.pdf]

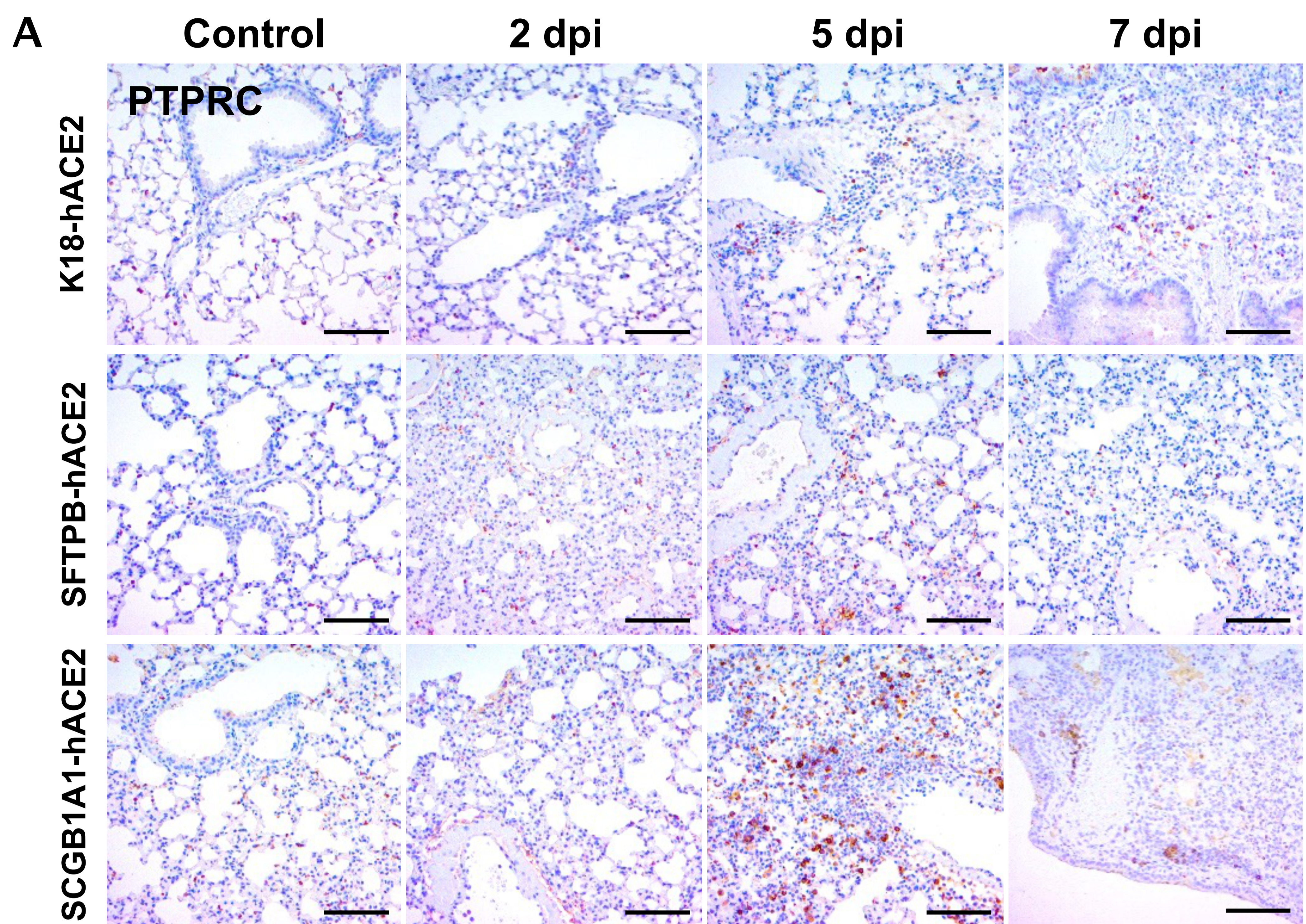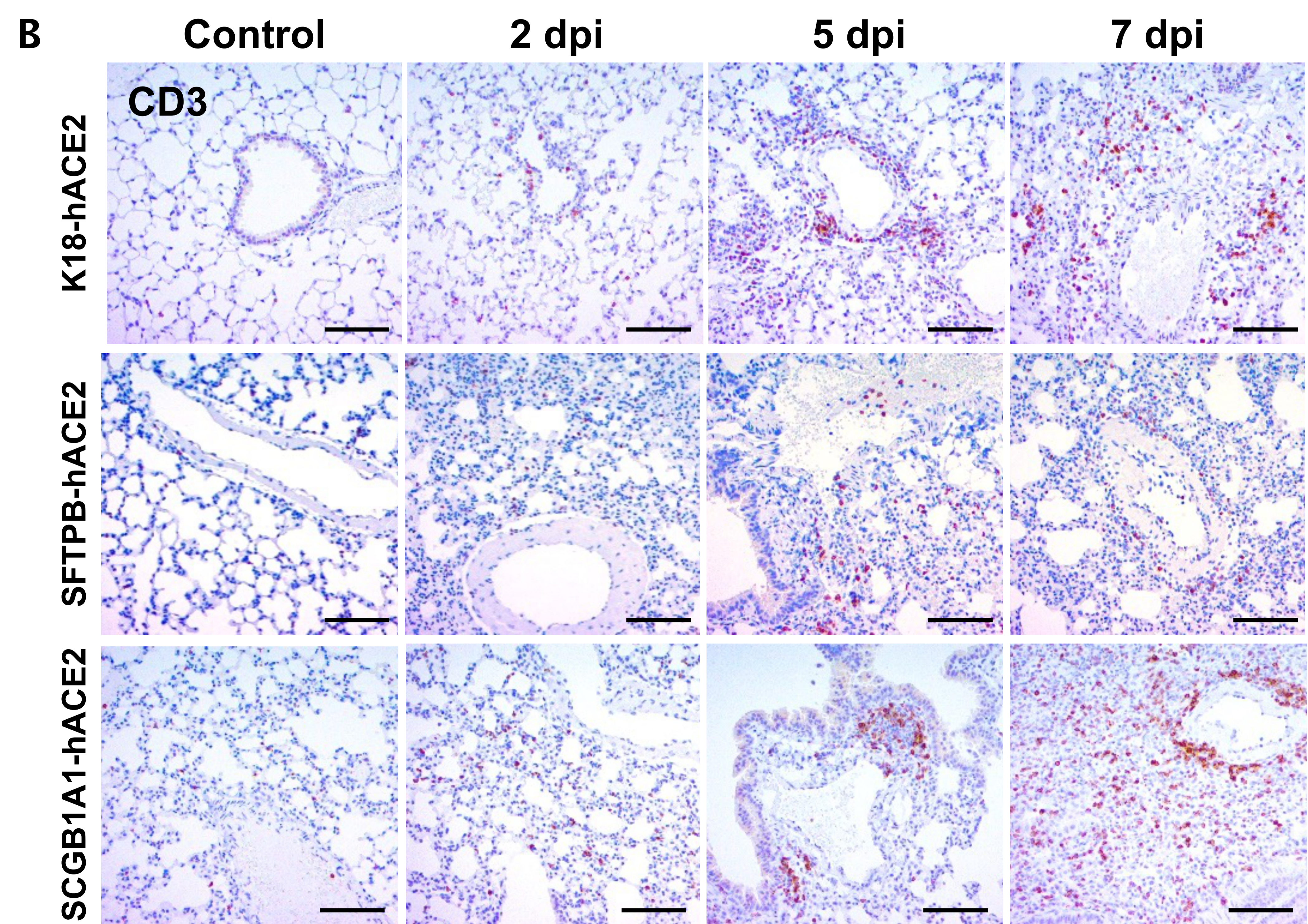

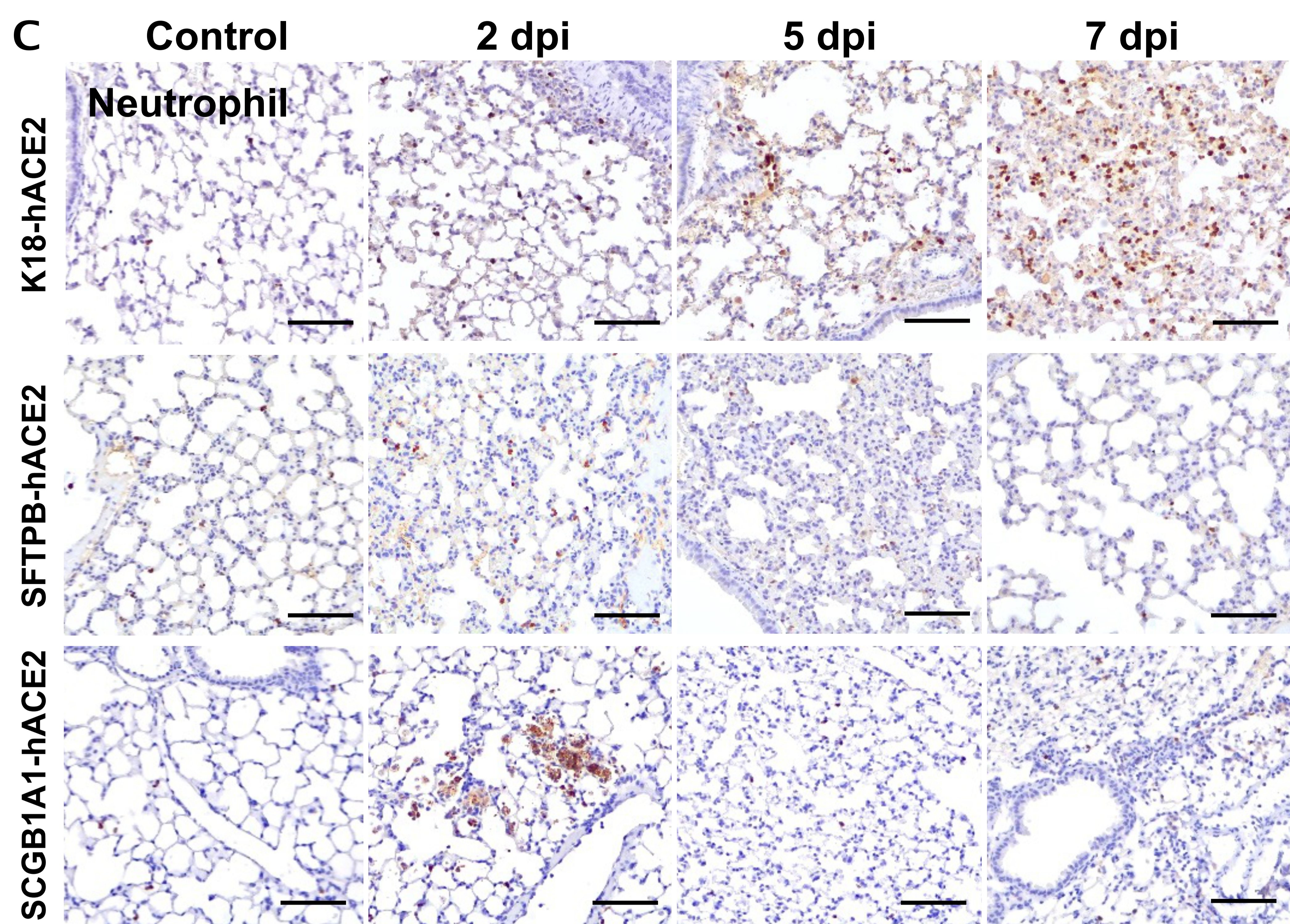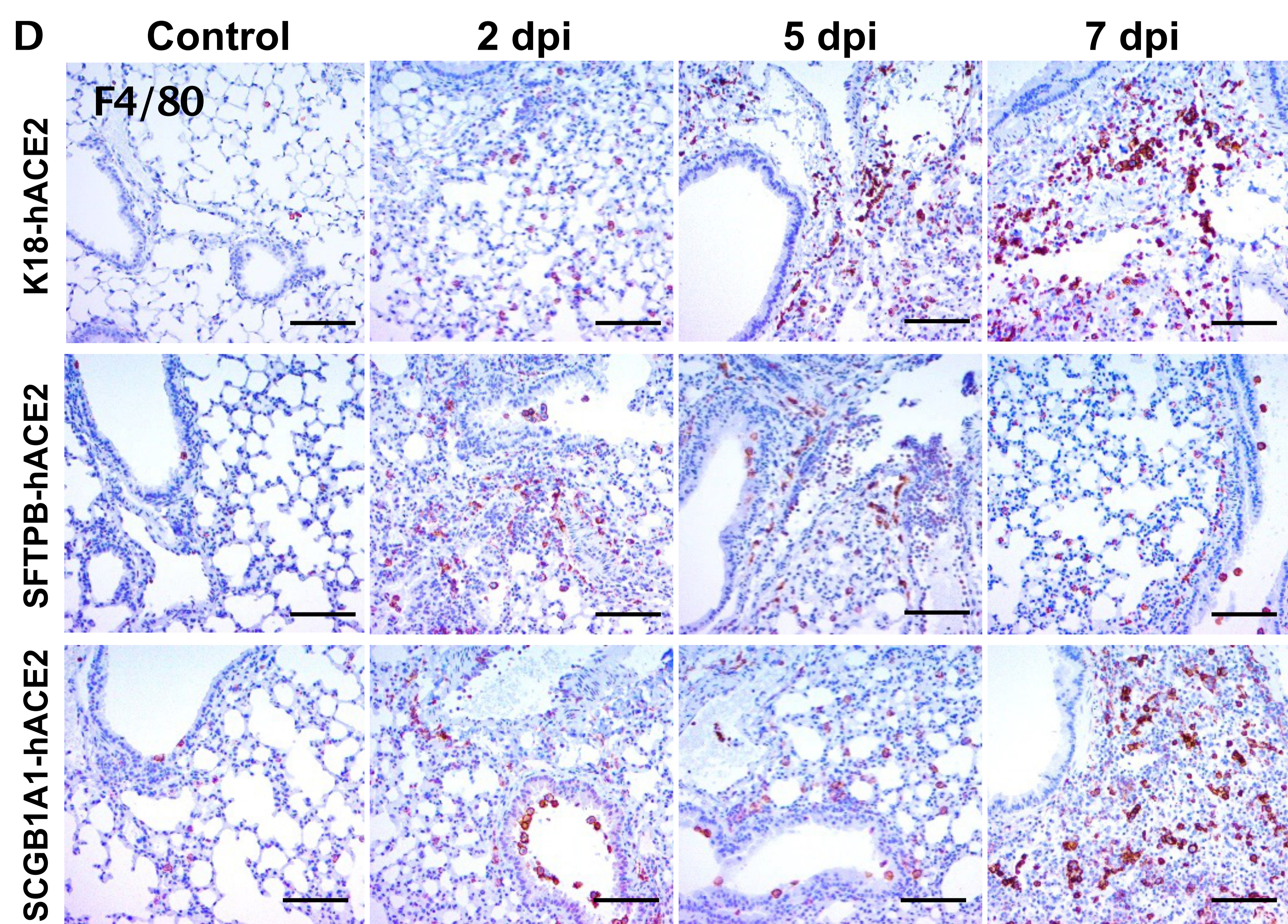

Supplement: Supplementary Figure 4 — Immune cell infiltration and distribution in the SARS-CoV-2-infected lungs of K18-hACE2, SFTPB-hACE2, and SCGB1A1-hACE2 mice. PTPRC+ cells (A), CD3+ cells (B), neutrophil (C), and F4/80+ macrophage (D) distribution in the lungs confirmed by immunohistochemistry. Each marker is stained brown and counterstained by DAPI (blue). [file Image_4.pdf]

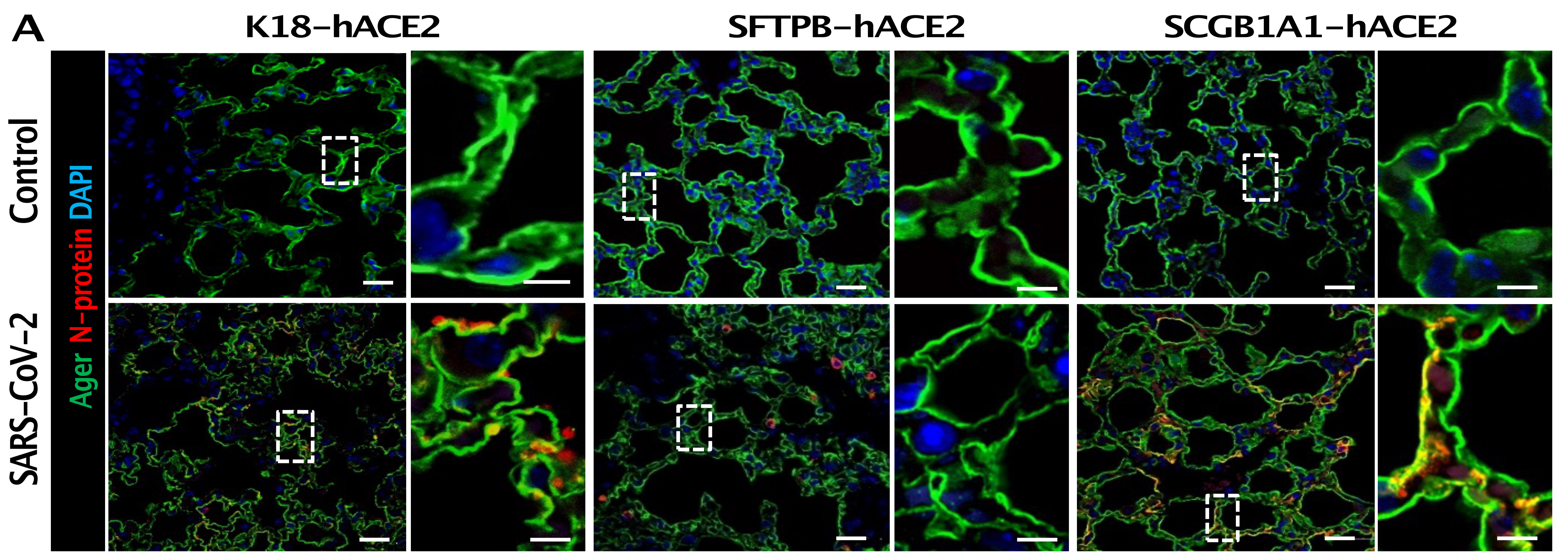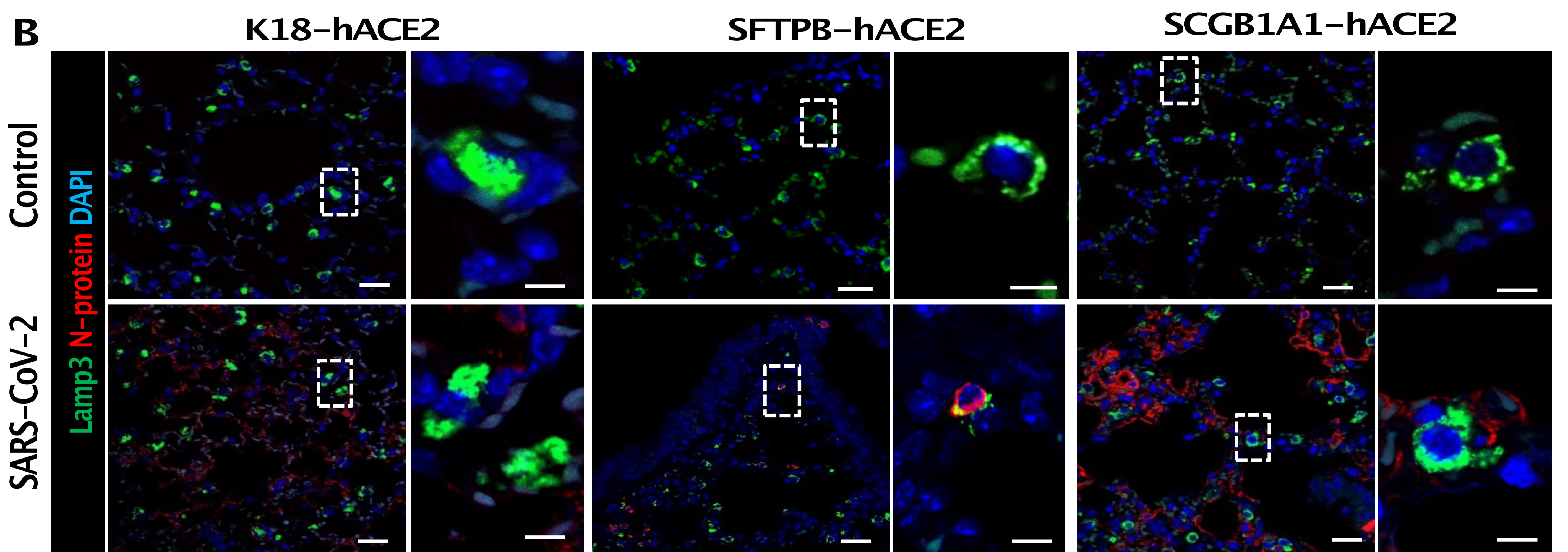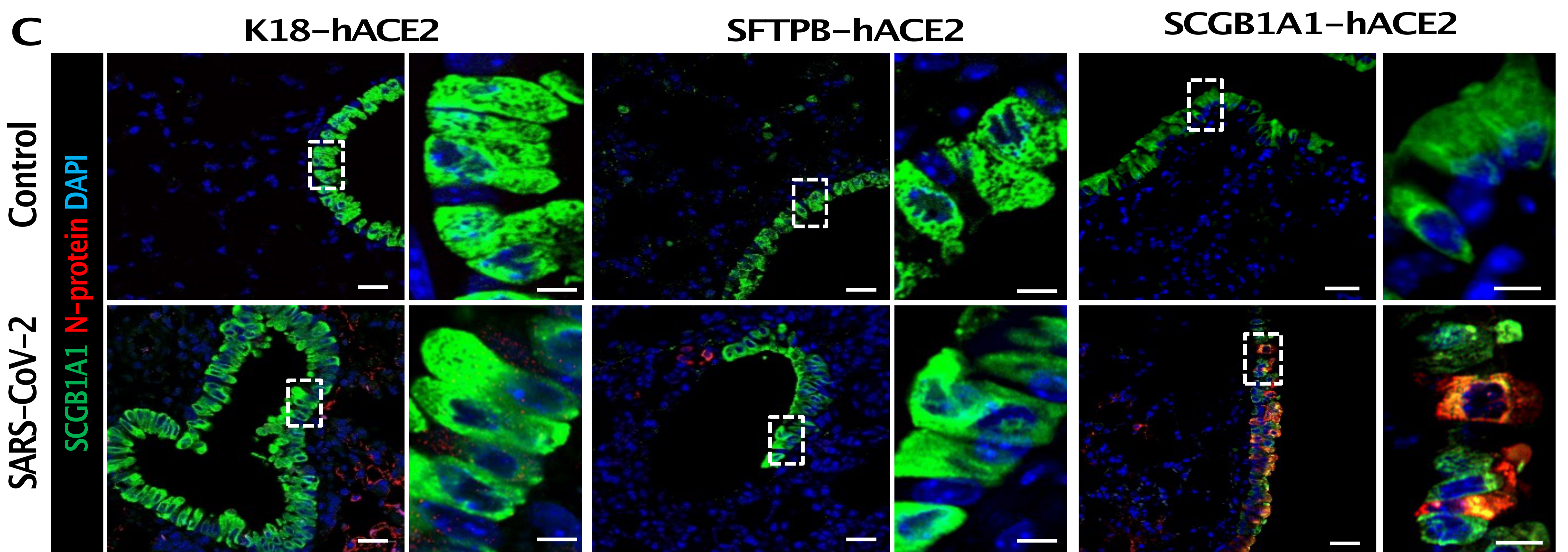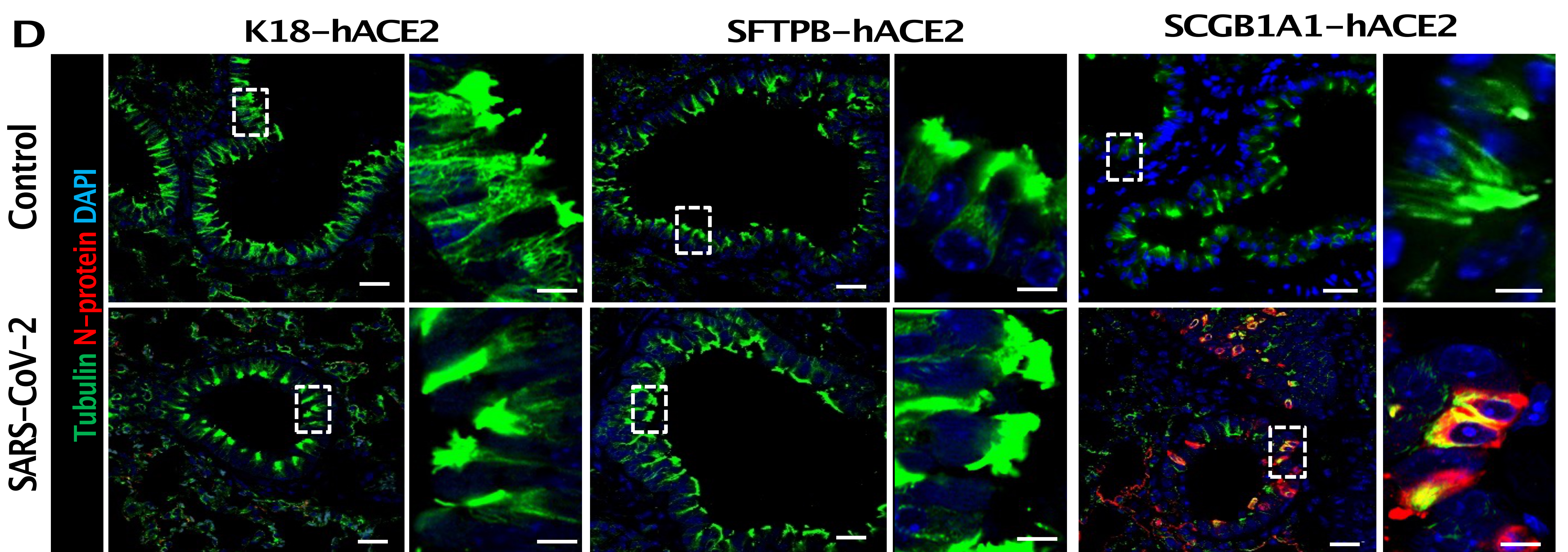

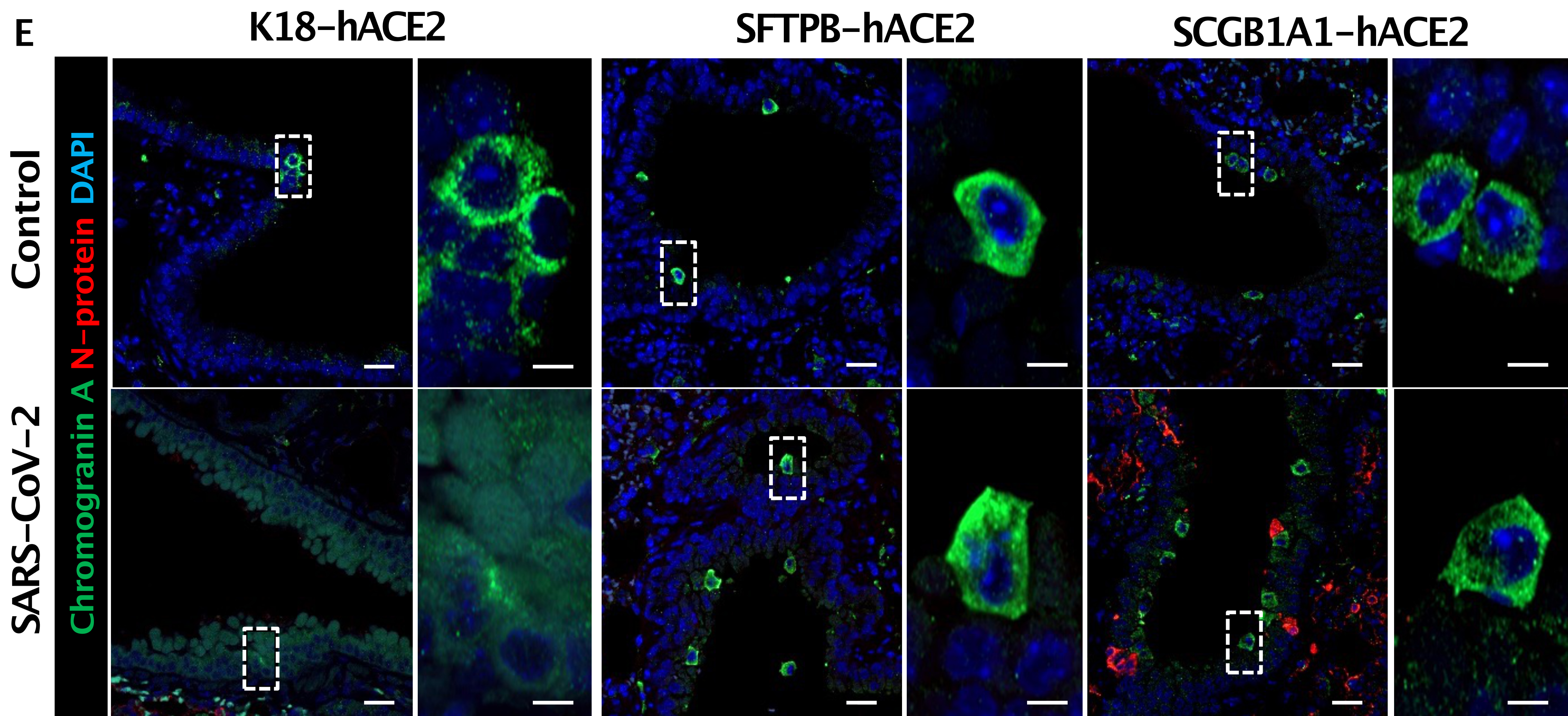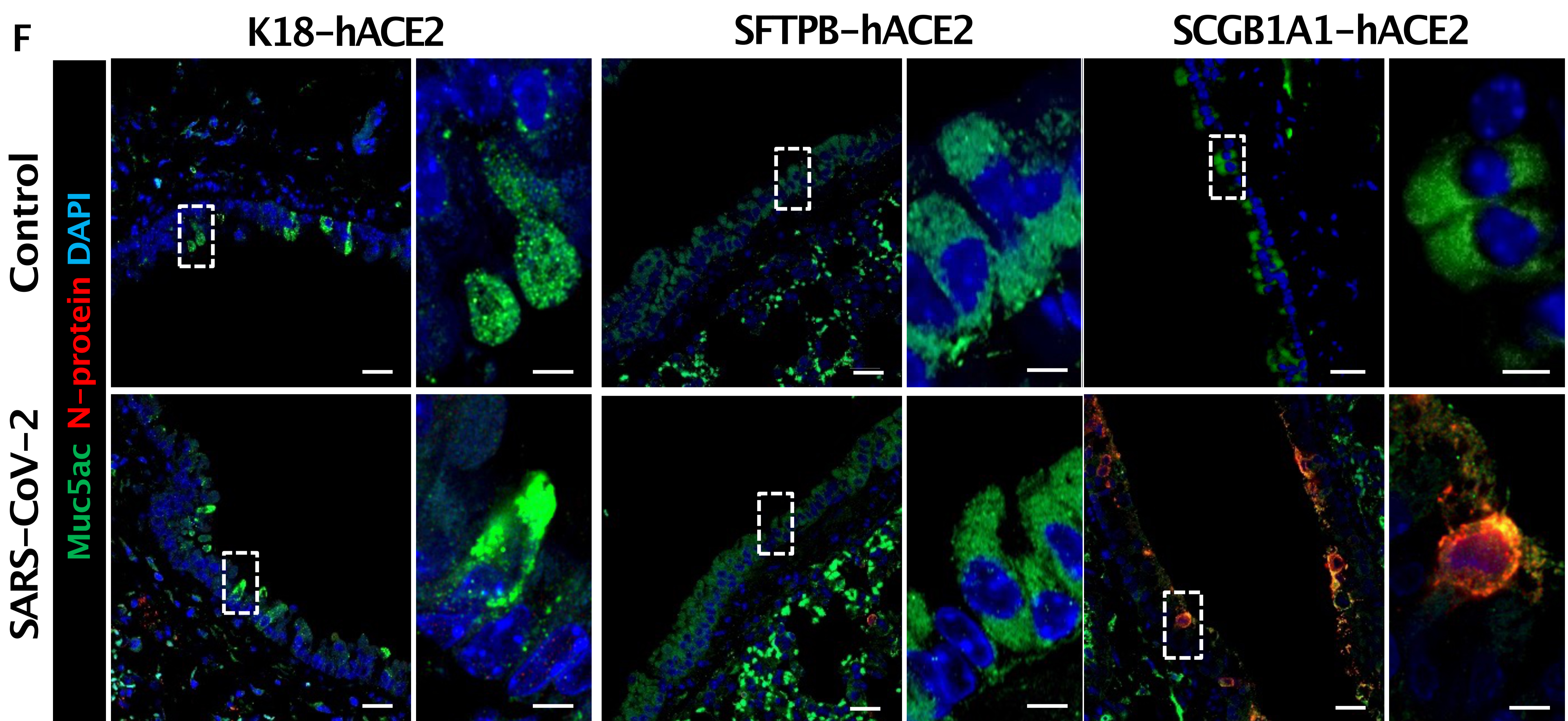

Supplement: Supplementary Figure 5 — Immunohistochemistry analysis of SARS-CoV-2 infectious target lung cells in K18-hACE2, SFTPB-hACE2, and SCGB1A1-hACE2 mice. N protein of SARS-CoV-2 (red) and lung cell type markers are double stained: (A) type I alveolar cell marker AGER, (B) type II alveolar cell marker LAMP3, (C) club cell marker SCGB1A1, (D) ciliated cell marker acetylated alpha-tubulin, (E) pulmonary neuroendocrine cell marker chromogranin A, and (F) epithelial goblet cell marker MUC5AC. [file Image_5.pdf]

A

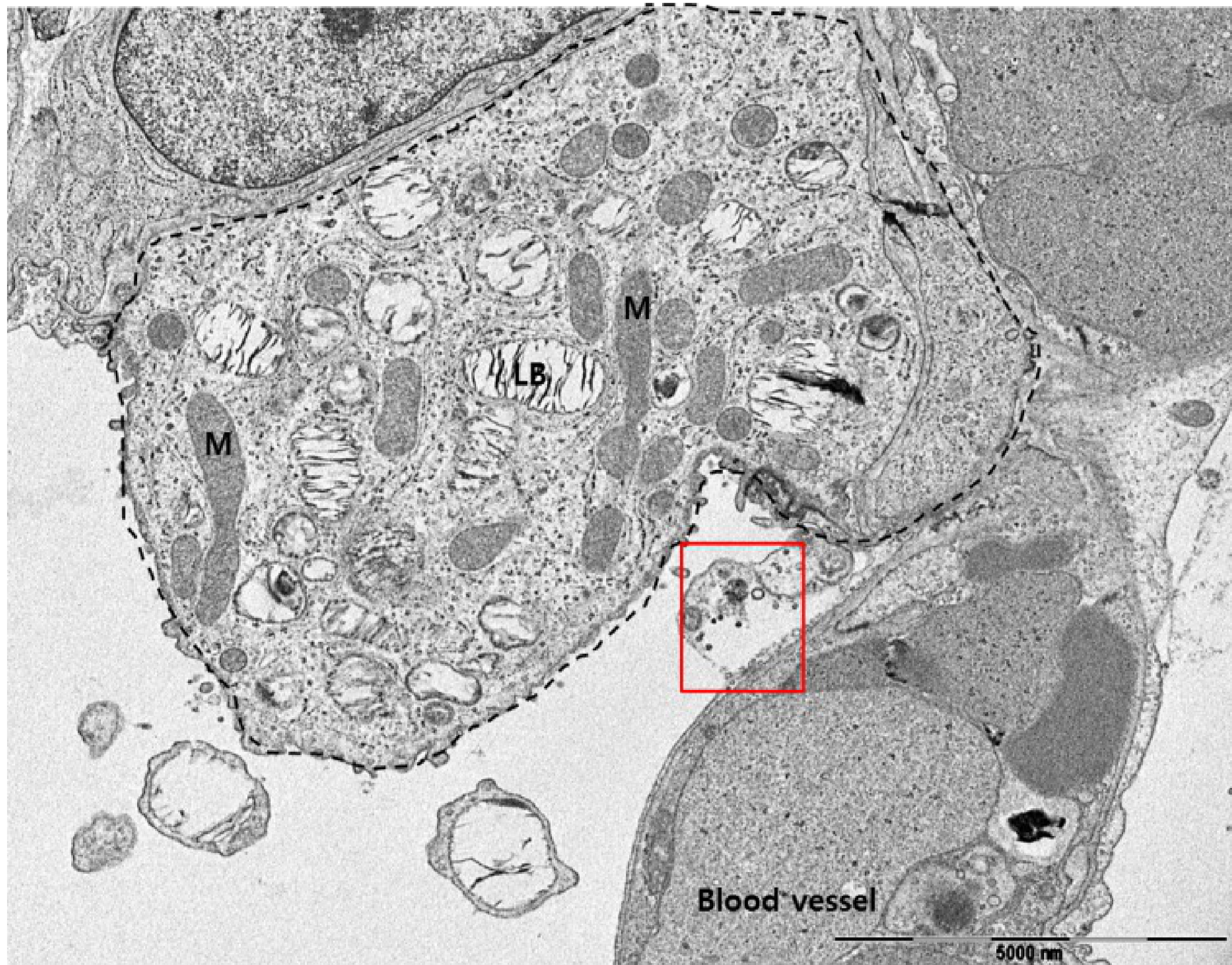

B

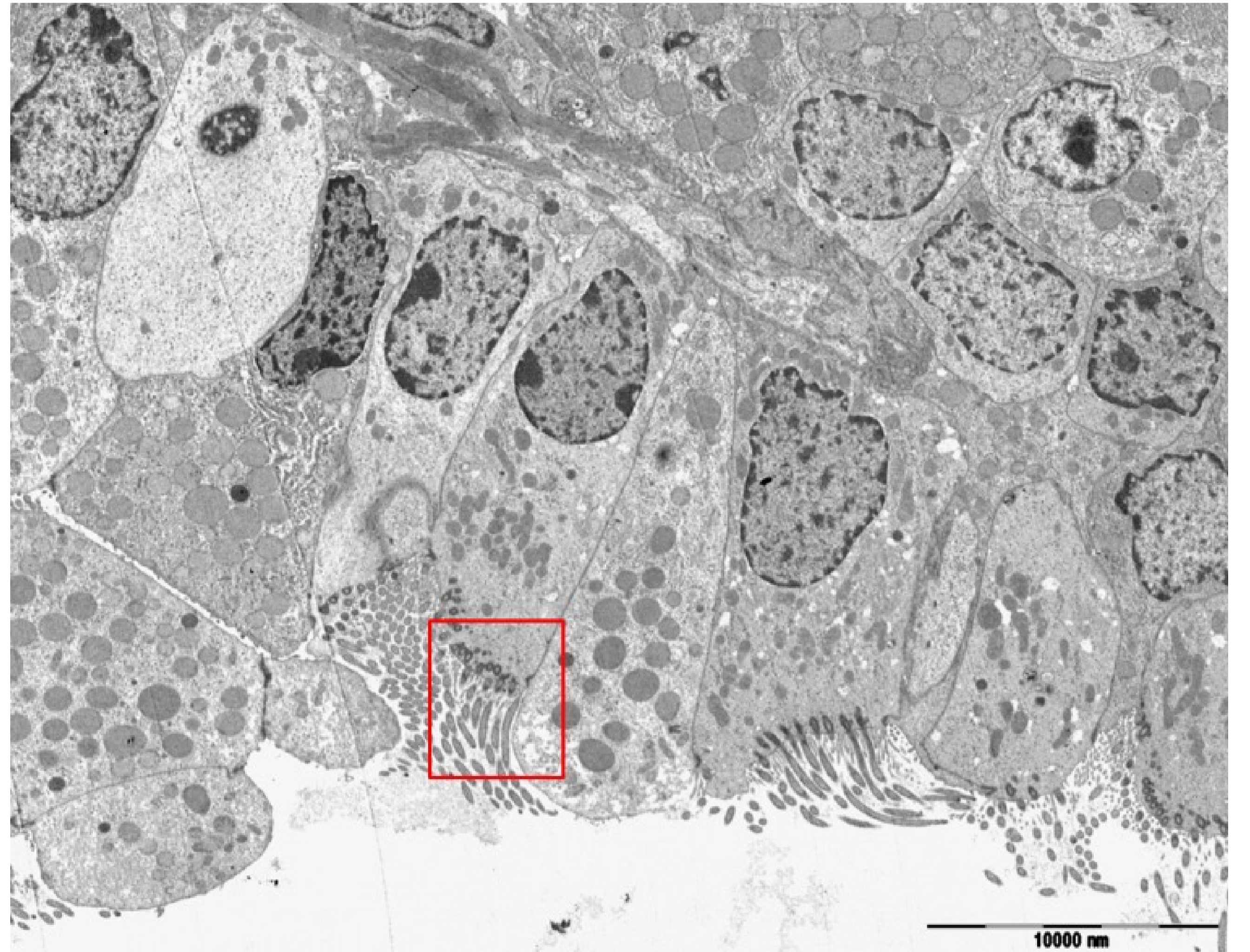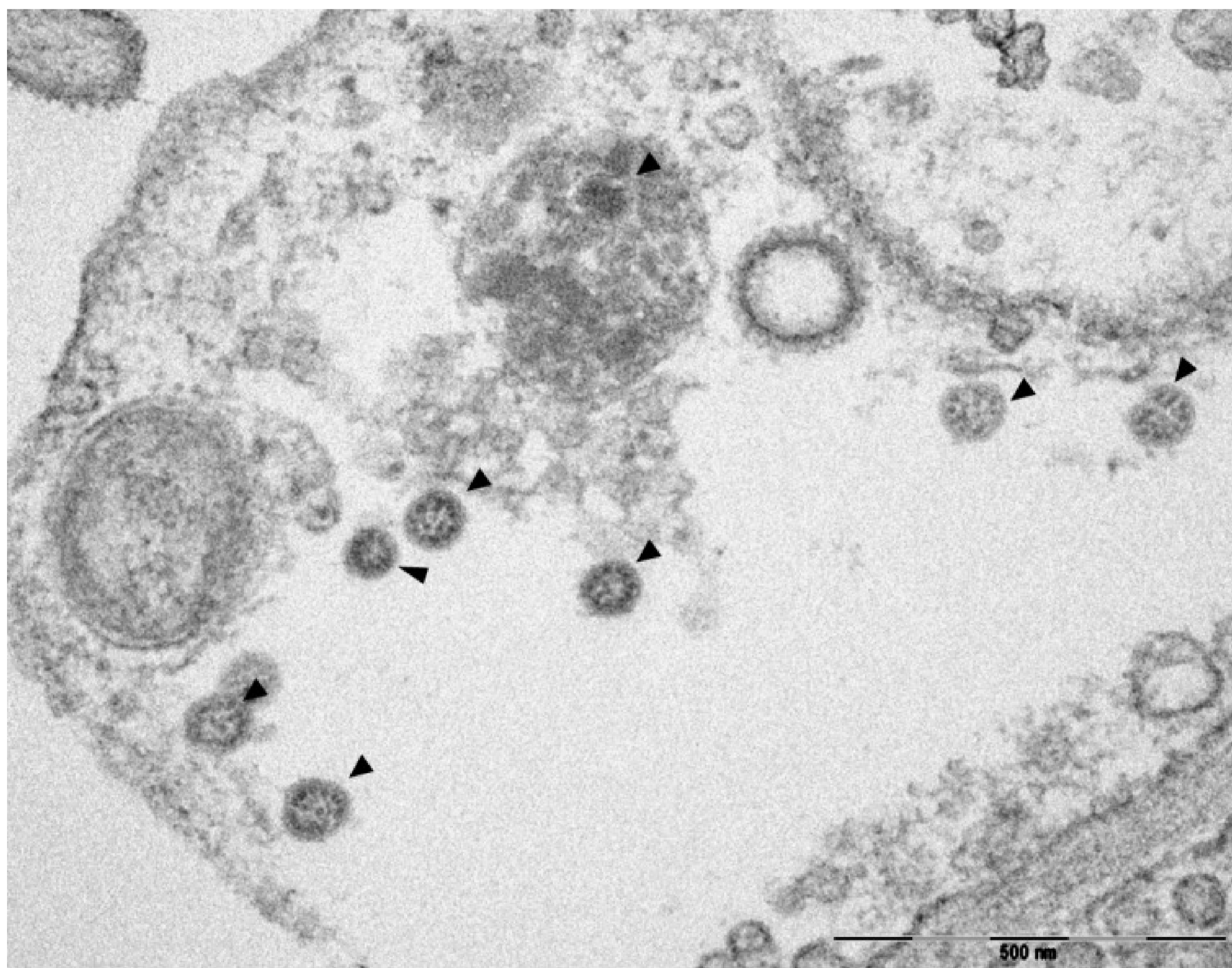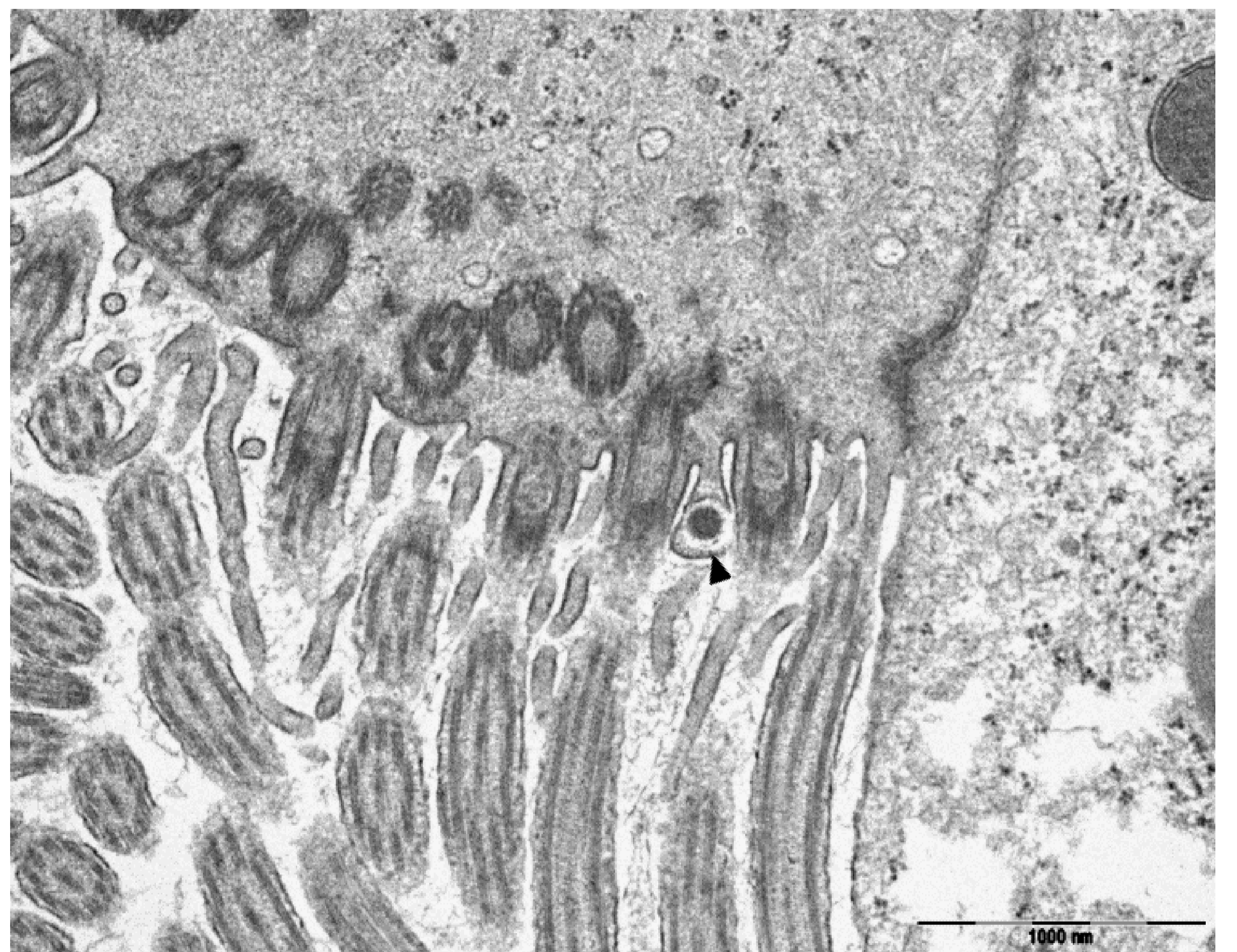

Supplement: Supplementary Figure 6 — SARS-CoV-2 particles detected in the lungs of K18-hACE2 and SCGB1A1-hACE2 mice. SARS-CoV-2 particles detected by electron microscopy. In K18-hACE2 mice, viral particles (black arrow heads) were distributed in type I alveolar pneumocytes (A). The dotted line is the type II alveolar pneumocyte. M, mitochondria; LB, lamellar body. In SCGB1A1-hACE2 mice, SARS-CoV-2 is bound to the cilia in bronchos (B). [file Image_6.pdf]

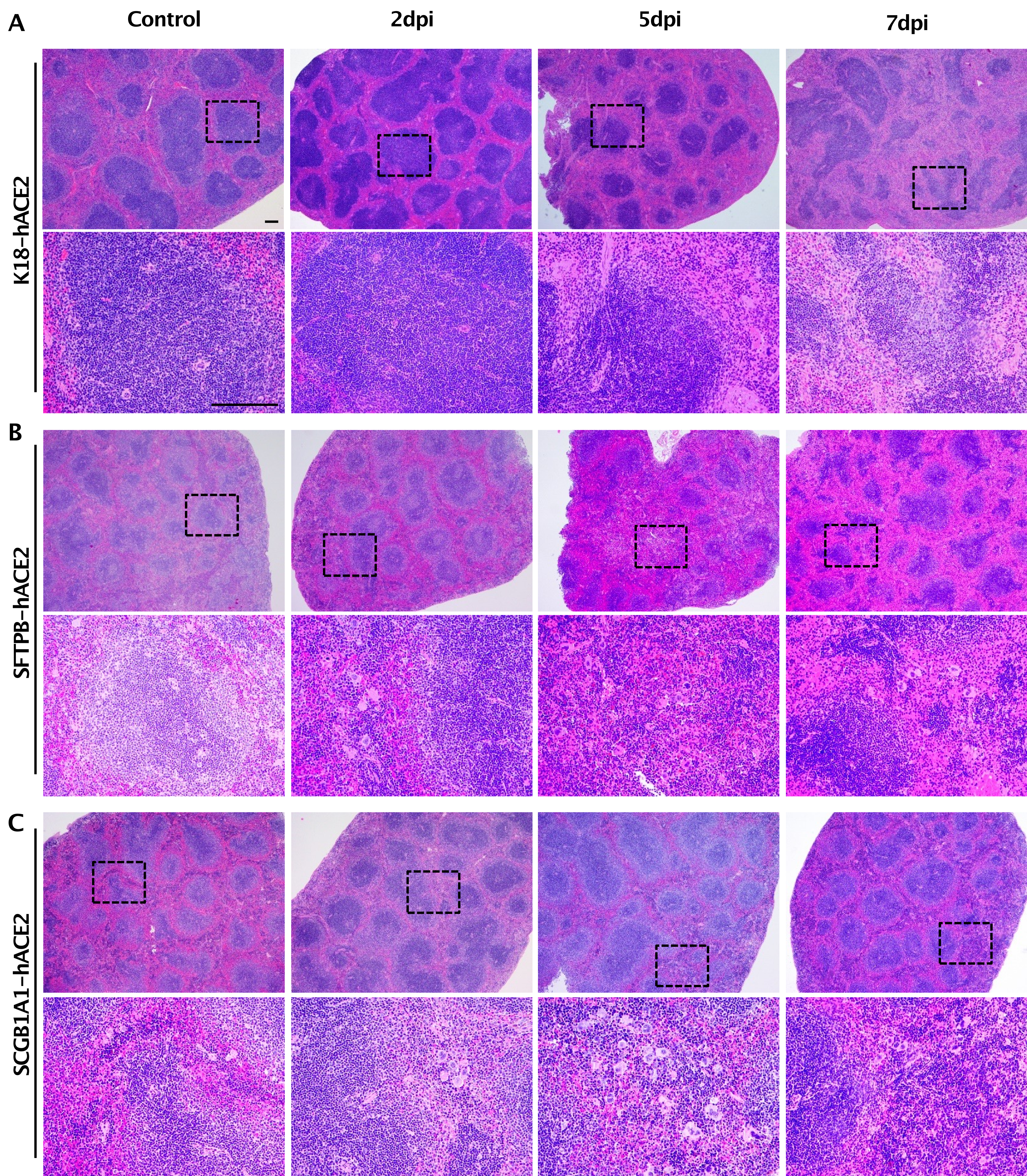

Supplement: Supplementary Figure 7 — Histopathological analysis of the spleen in SARS-CoV-2-infected K18-hACE2, SFTPB-hACE2, and SCGB1A1-hACE2 mice. (A–C) Pathological analysis of the spleen following intranasal infection of 1 × 10^5^ PFU SARS-CoV-2 in K18-hACE2 (A), SFTPB-hACE2 (B), and SCGB1A1-hACE2 (C) mice. Autopsy was conducted at 1, 2, 5, and 7 dpi. The scale bars are 100 μm (left panels) and 50 μm (right panels). [file Image_7.pdf]

K18-hACE2

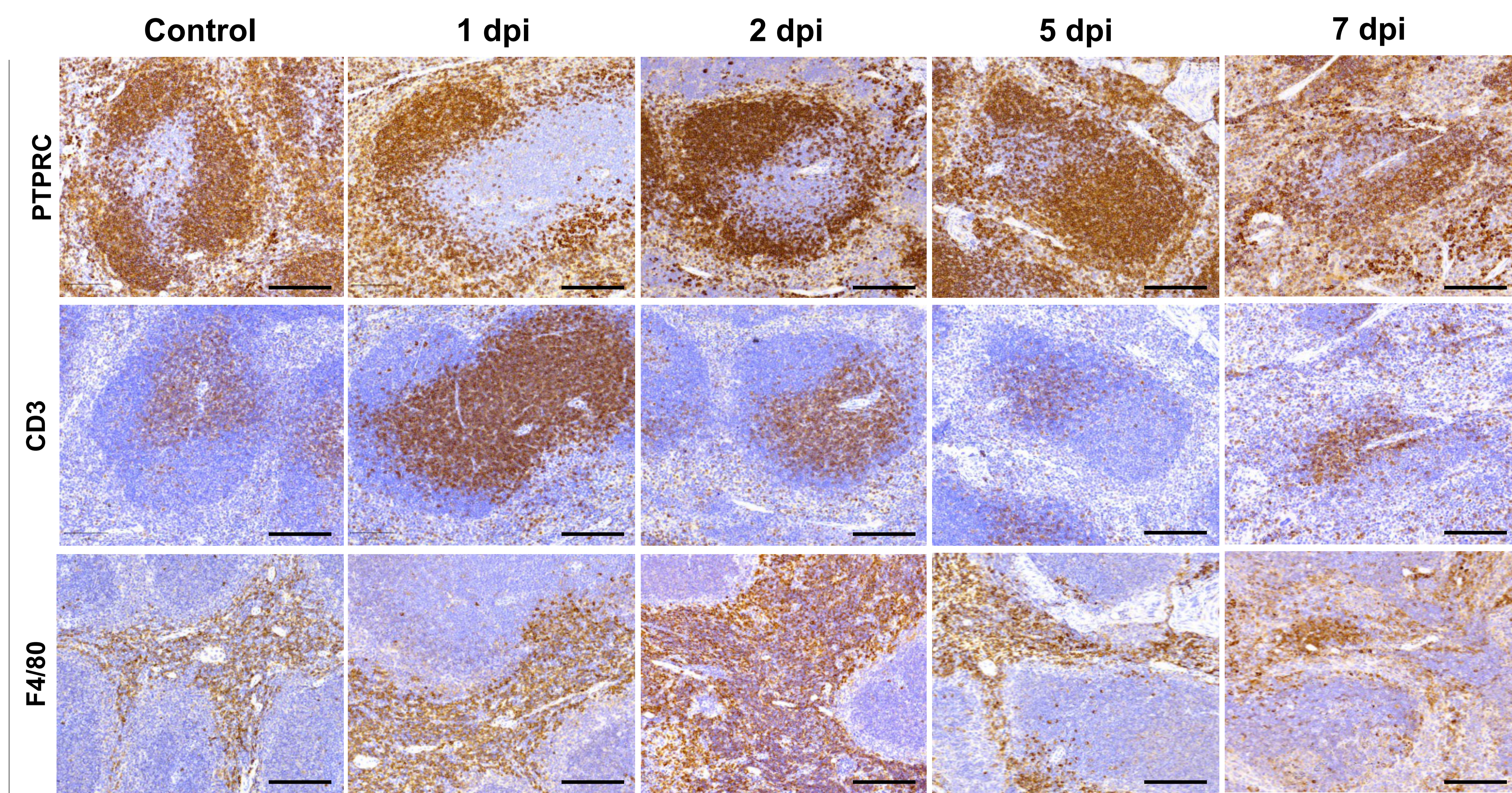

Supplement: Supplementary Figure 8 — Immune cell distribution in the SARS-CoV-2-infected spleen of K18-hACE2 mice. The B cell marker PTPRC, T cell marker CD3, and macrophage marker F4/80 were stained in SARS-CoV-2-infected K18-hACE2 mice. [file Image_8.pdf]

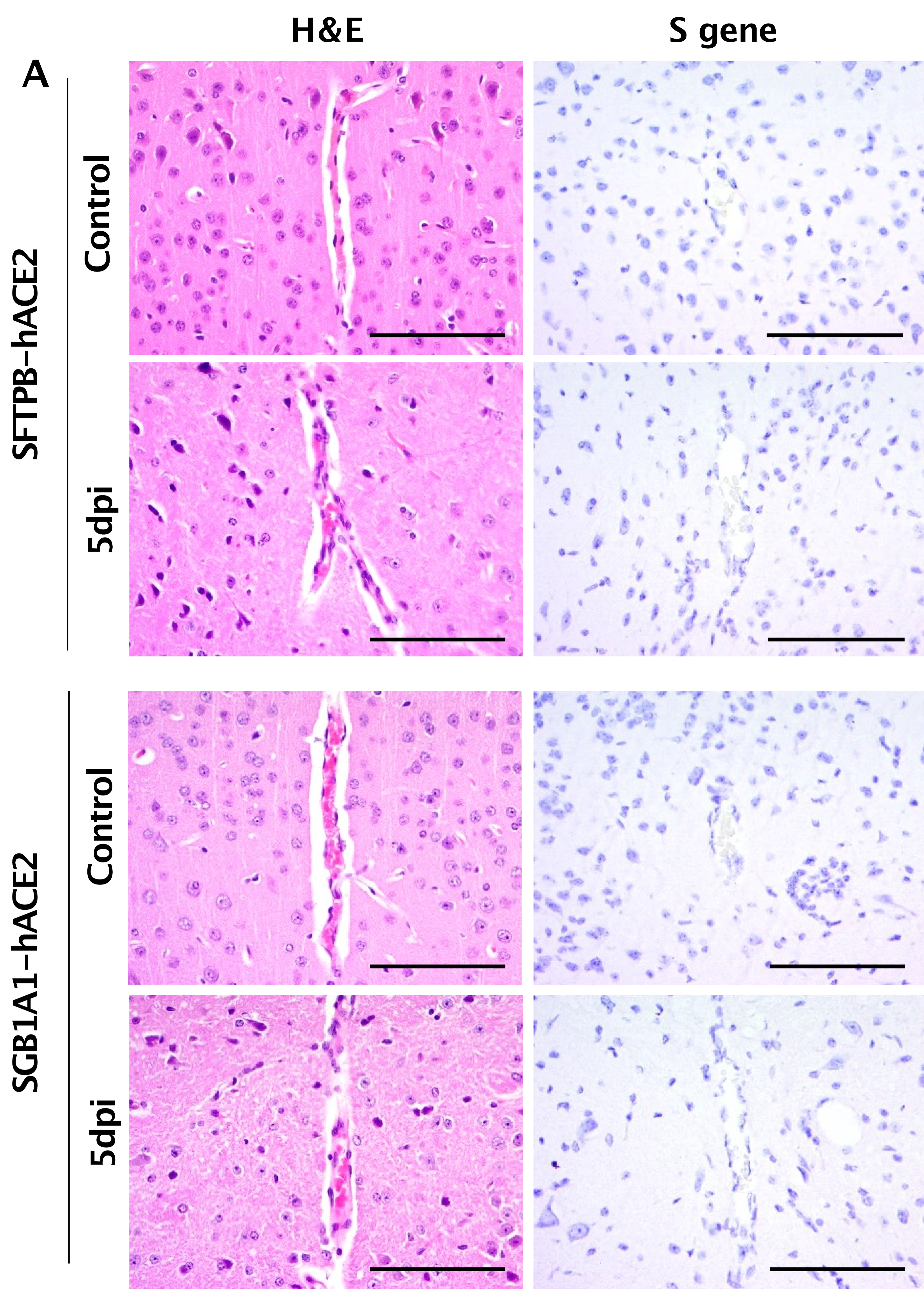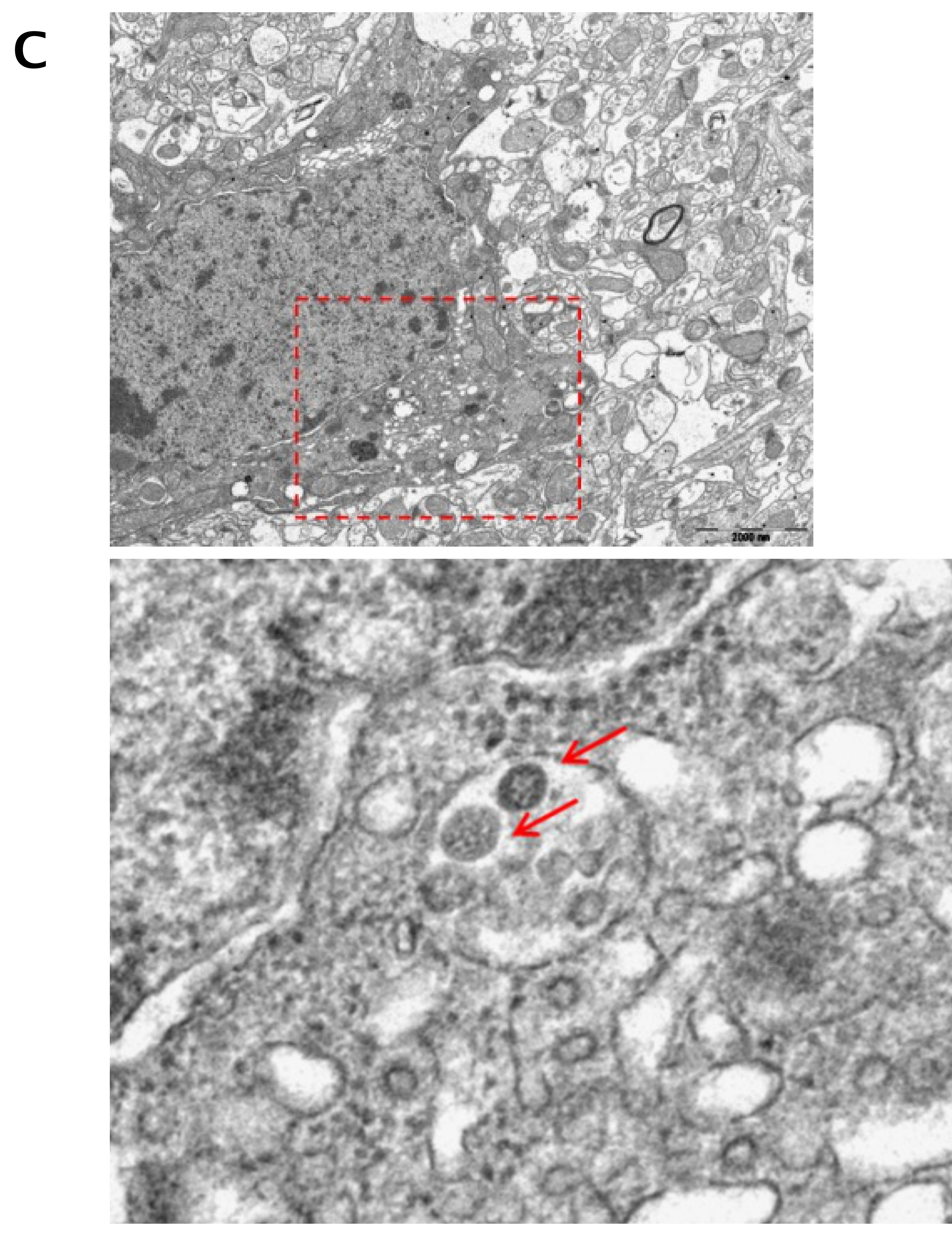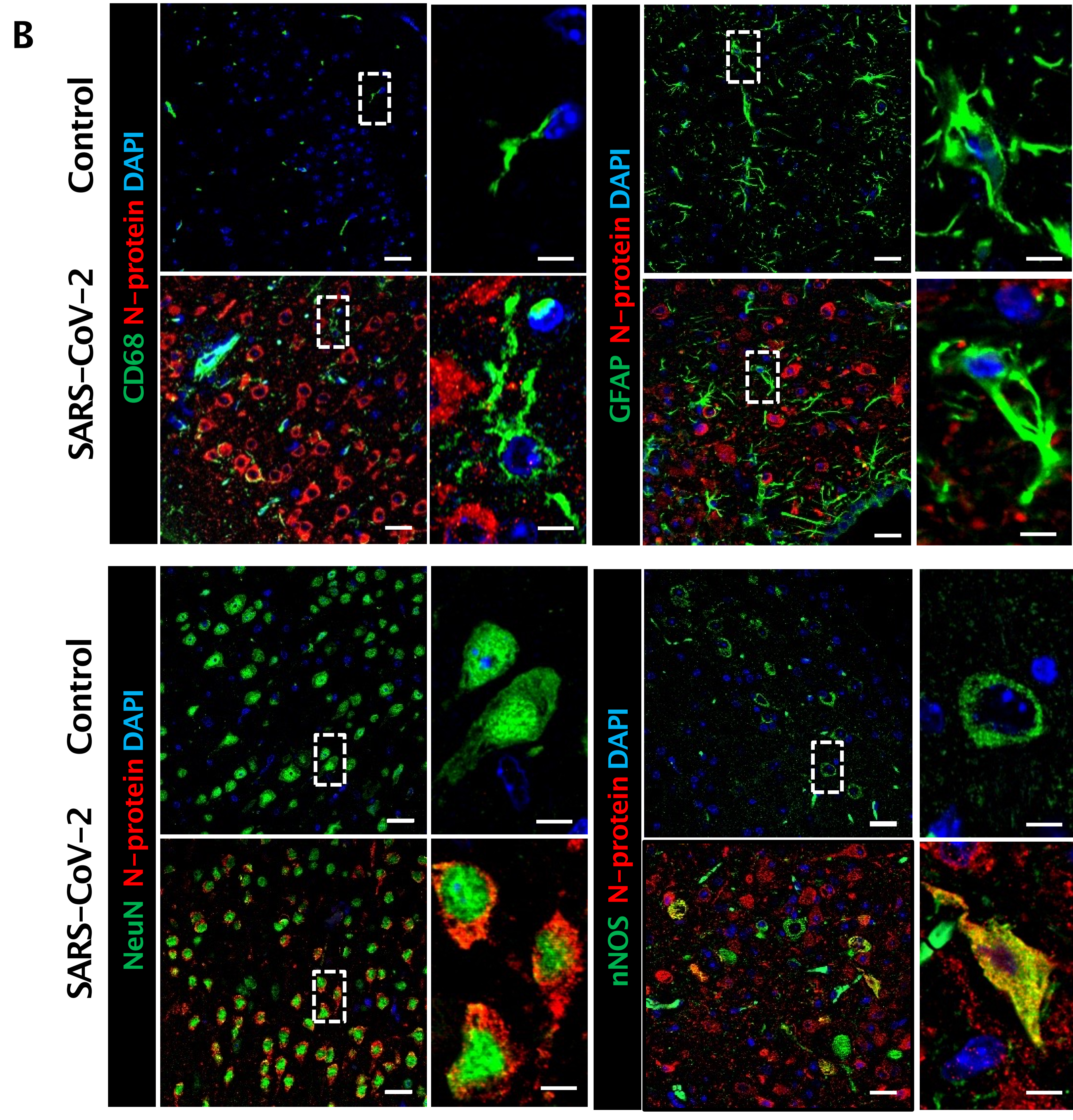

Supplement: Supplementary Figure 9 — Lesion and infectious target cells in the brain of SARS-CoV-2-infected SFTPB-hACE2 and SCGB1A1-hACE2 mice. Brain lesion, diagnosed as perivesicular coffing, was not detected in the brains of SFTPB-hACE2, SCGB1A1-hACE2, and control mice at 5 dpi (A). SARS-CoV-2 infectious target cells in the brain of K18-hACE2 mice (B). SARS-CoV-2 viral particles were detected in K18-hACE2 mice (red arrow) (C). [file Image_9.pdf]
