# Supplementary material for: Mouse models of lung-specific SARS-CoV-2 infection with moderate pathological traits
Source: Front Immunol. 2022 Nov 15;13:1055811. doi: 10.3389/fimmu.2022.1055811 (PMC9706212; doi:10.3389/fimmu.2022.1055811)
Supplement: Supplementary Figure 3 — Histopathological analysis of the SARS-CoV-2-infected lung in K18-hACE2, SFTPB-hACE2, and SCGB1A1-hACE2 mice. (A–C) H&E staining of the lung following intranasally infection of 1 × 10^5^ PFU SARS-CoV-2 in K18-hACE2 (A), SFTPB-hACE2 (B), and SCGB1A1-hACE2 (C) mice. Autopsy was conducted at 1, 2, 5, and 7 dpi. The scale bars are 100 μm (left panels) and 50 μm (right panels). [file Image_3.pdf]

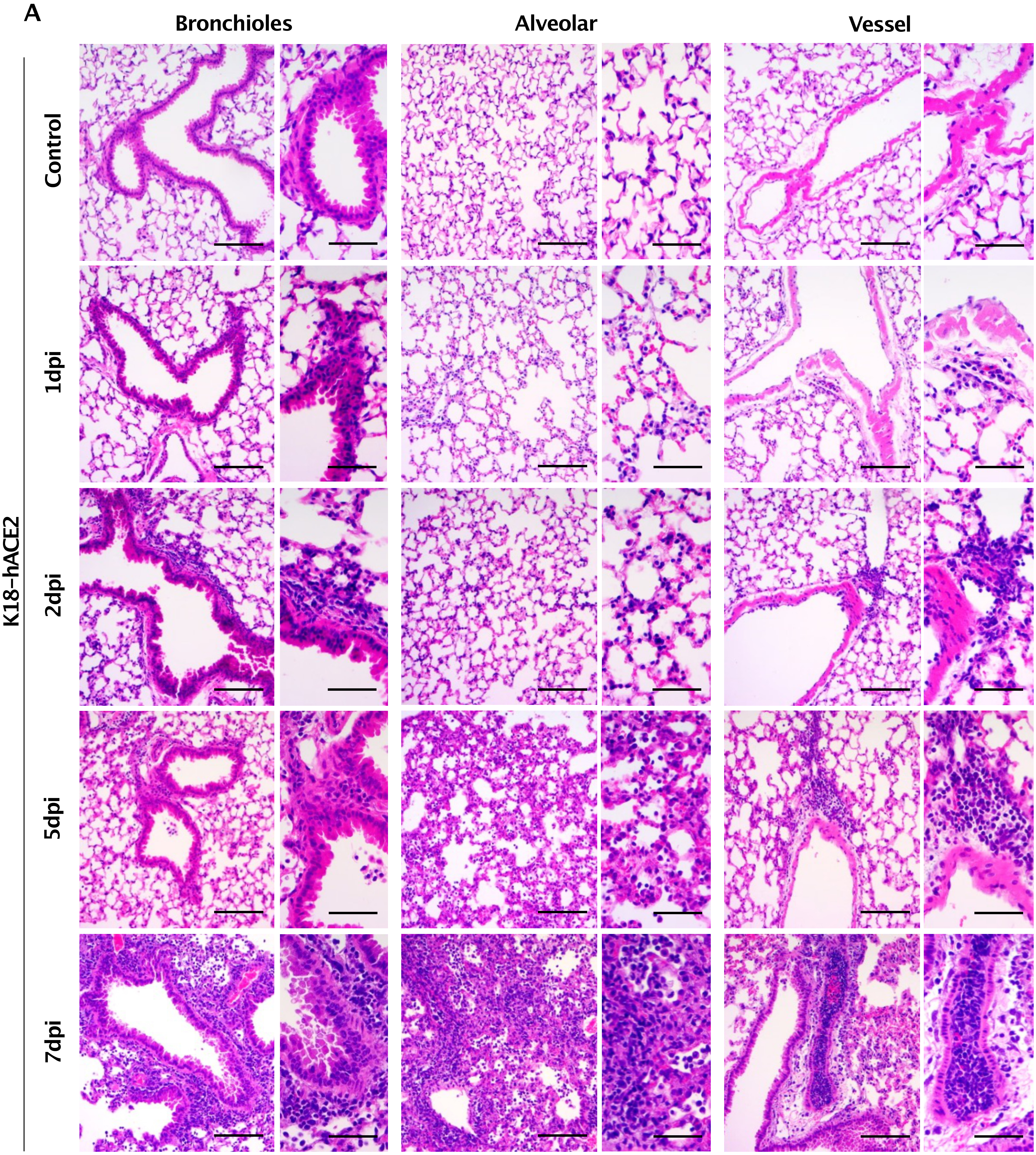
A
Bronchioles
Alveolar
Vessel
K18-hACE2
Control
1dpi
2dpi
5dpi
7dpi

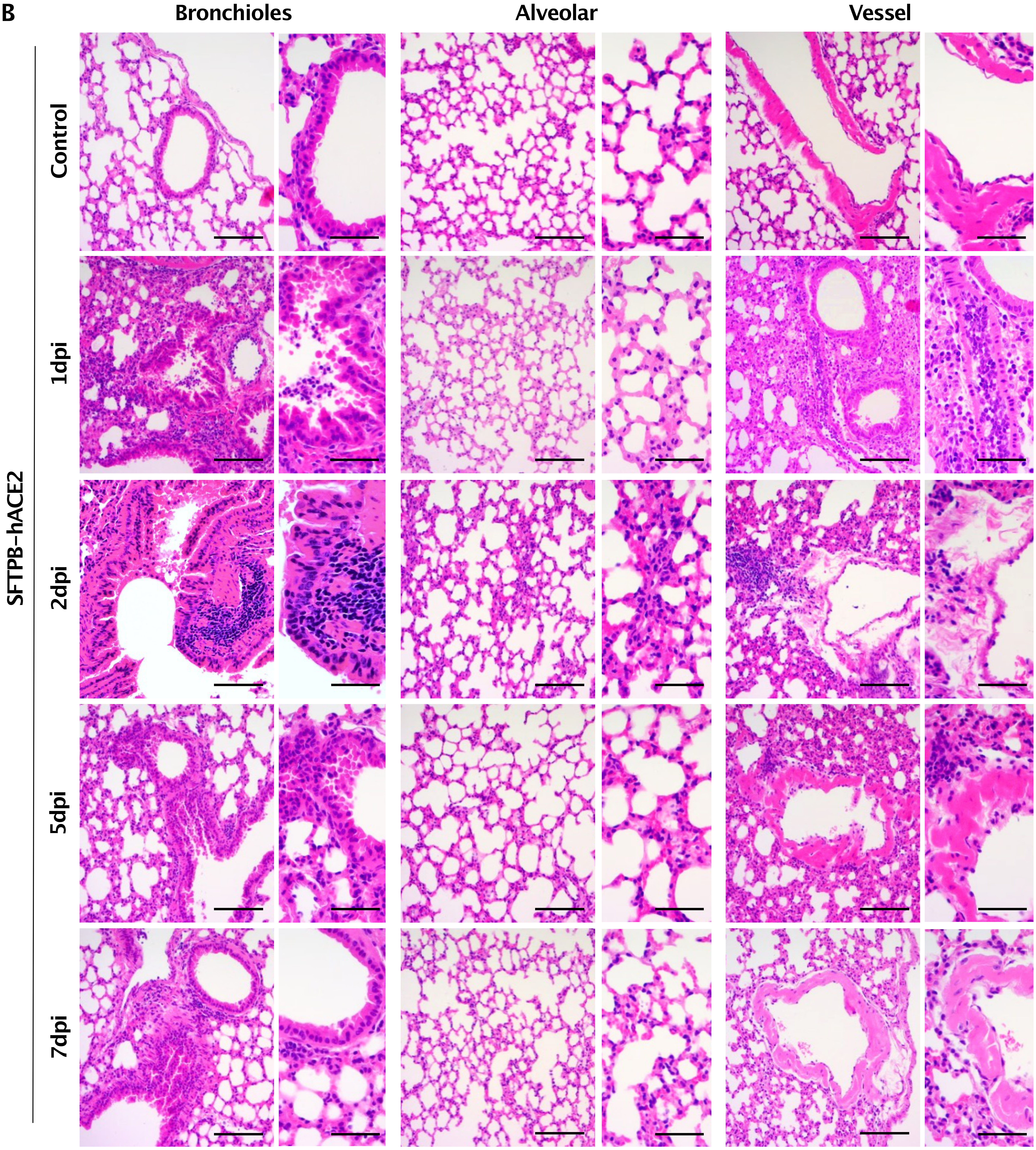
B
Bronchioles
Alveolar
Vessel
SFTPB-hACE2
Control
1dpi
2dpi
5dpi
7dpi

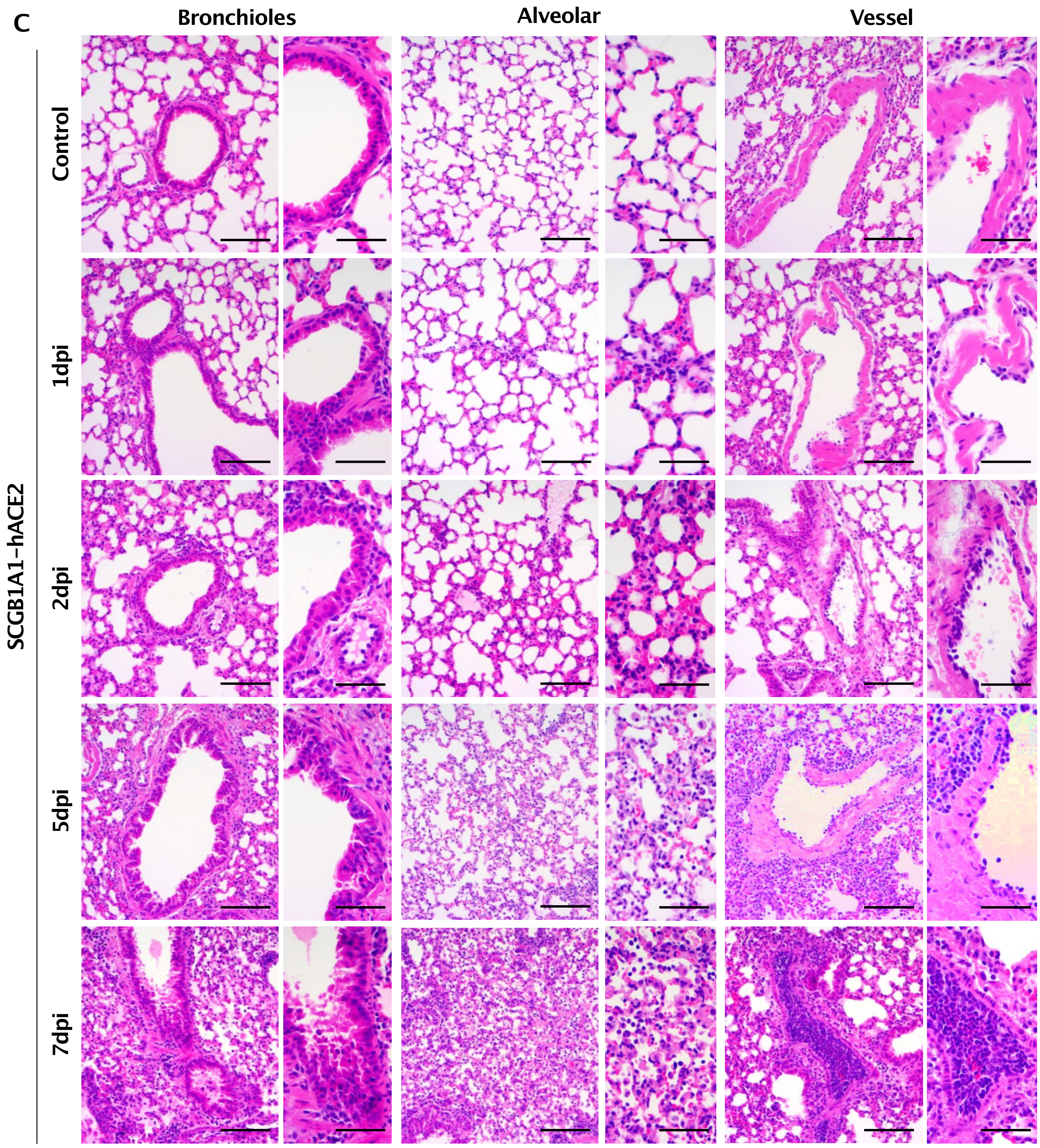
C
Bronchioles
Alveolar
Vessel
SCGB1A1-hACE2
Control
1dpi
2dpi
5dpi
7dpi
